# Synthesis and Characterization of a “Clickable” PBR28 TSPO-Selective Ligand Derivative Suitable for the Functionalization of Biodegradable Polymer Nanoparticles

**DOI:** 10.3390/nano11071693

**Published:** 2021-06-28

**Authors:** Renato Auriemma, Mattia Sponchioni, Umberto Capasso Palmiero, Giacomo Rossino, Arianna Rossetti, Andrea Marsala, Simona Collina, Alessandro Sacchetti, Davide Moscatelli, Marco Peviani

**Affiliations:** 1 Department of Chemistry, Materials and Chemical Engineering “Giulio Natta”, Politecnico di Milano, Via Mancinelli 7, 20131 Milano, Italy; renato.auriemma@polimi.it (R.A.); arianna.rossetti@polimi.it (A.R.); alessandro.sacchetti@polimi.it (A.S.); davide.moscatelli@polimi.it (D.M.); 2 Department of Chemistry and Applied Biosciences, ETH Zürich, Vladimir-Prelog-Weg 1-5/10, 8093 Zürich, Switzerland; umberto.capasso@chem.ethz.ch; 3 Department of Drug Sciences, University of Pavia, Via Taramelli 12, 27100 Pavia, Italy; giacomo.rossino01@universitadipavia.it (G.R.); simona.collina@unipv.it (S.C.); 4 Department of Biology and Biotechnology “L. Spallanzani”, University of Pavia, Via Ferrata 9, 27100 Pavia, Italy; andrea.marsala01@universitadipavia.it; 5 Gene Therapy Program, Dana Farber/Boston Children’s Cancer and Blood Disorders Center, 450 Brookline Ave., Boston, MA 02215, USA; 6 Harvard Medical School, Boston, MA 02115, USA

**Keywords:** neuroinflammation, microglia, TSPO, active targeting, click chemistry, polymer nanoparticles

## Abstract

Reactive microgliosis is a pathological hallmark that accompanies neuronal demise in many neurodegenerative diseases, ranging from acute brain/spinal cord injuries to chronic diseases, such as amyotrophic lateral sclerosis (ALS), Alzheimer’s disease (AD) and age-related dementia. One strategy to assess and monitor microgliosis is to use positron emission tomography (PET) by exploiting radioligands selective for the 18 kDa translocator protein (TSPO) which is highly upregulated in the brain in pathological conditions. Several TSPO ligands have been developed and validated, so far. Among these, PBR28 has been widely adopted for PET imaging at both preclinical and clinical levels, thanks to its high brain penetration and high selectivity. For this reason, PBR28 represents a good candidate for functionalization strategies, where this ligand could be exploited to drive selective targeting of TSPO-expressing cells. Since the PBR28 structure lacks functional moieties that could be exploited for derivatization, in this work we explored a synthetic pathway for the synthesis of a PBR28 derivative carrying an alkyne group (PBR-alkyne), enabling the fast conjugation of the ligand through azide-alkyne cycloaddition, also known as click-chemistry. As a proof of concept, we demonstrated in silico that the derivatized PBR28 ligand maintains the capability to fit into the TSPO binding pocked, and we successfully exploited PBR-alkyne to decorate zwitterionic biodegradable polymer nanoparticles (NPs) resulting in efficient internalization in cultured microglia-like cell lines.

## 1. Introduction

Assessment of microglial activation in neurodegenerative conditions can be performed through neuroimaging of the 18 kDa translocator protein (TSPO) using selective TSPO radioligands which allow the exploration of microgliosis in neurological disorders [1,2,3]. TSPO, formerly named peripheral benzodiazepine receptor [4,5], is part of a multimeric protein complex associated with the outer mitochondrial membrane. TSPO is highly expressed in the adrenals, kidneys, lungs and spleen. It is hardly detectable in the healthy brain whereas it becomes overexpressed in activated glial cells. Microglia cells are the main source of TSPO in the CNS; some reports identified TSPO also in hypertrophic astrocytes [6] and endothelial cells. However, the localization of TSPO in these cell types is still debated. TSPO is proposed as a useful marker to monitor neuroinflammation in the brain. Interestingly, increased expression of TSPO was reported also in several cancer cell lines as well as in tumor-associated macrophages, suggesting that TSPO could play a role also in tumorigenesis. Altogether, these observations prompted the attempts to exploit TSPO-selective ligands for several applications, including (i) development of radioligands for PET imaging studies [1,3,7]; (ii) conjugation with small molecule therapeutics to achieve targeted delivery of anticancer drugs [8]; (iii) functionalization of nanoparticles (NPs) to achieve cell-selective internalization [9].

The first generation of TSPO-selective high-affinity ligands comprised Ro5-4864 and PK11195 [2]. Despite their ability to bind TSPO, these ligands displayed high non-specific background in PET studies, probably because of the entrapment in lipids-enriched tissues due to their highly lipophilic nature [2]. Therefore, second-generation ligands were generated, endowed with higher TSPO-receptor affinity and better in vivo pharmacokinetic profile. These molecules include PBR01 [10], PBR28 [10,11] and DAA1106 [12]. Among these ligands, PBR28 has been widely adopted for PET imaging studies, both at preclinical and clinical levels, thanks to its high brain penetration, high signal specificity and low non-specific binding. Despite these excellent properties, PBR28 lacks functional groups that could enable the conjugation with small molecule therapeutics or drug delivery vehicles for TSPO-targeted applications.

In this work, we designed a synthesis pathway that allowed us to generate a novel PBR28-derivative sharing the molecular structure of PBR28 but carrying an alkyne group enabling its facile conjugation with other compounds, via azide-alkyne cycloaddition, also known as click chemistry. In fact, this is a class of reactions well known for their occurrence at mild conditions, high yields and stereospecificity [13,14], which could streamline the production of armed TSPO-ligands.

To validate this concept, here we exploited the newly synthesized PBR28-derivative to decorate, via click chemistry, zwitterionic biodegradable polymer nanoparticles (NPs) modified with azide groups exposed on the surface [15,16,17] and demonstrated that this functionalization allows efficient internalization in microglial cells in vitro.

## 2. Materials and Methods

### 2.1. Materials

ε-Caprolactone (CL, 97% MW = 114.14 g/mol), Stannous Octoate (Sn(Oct)_2_, 92.5–100%, MW = 405.12 g/mol), 2-hydroxyethyl methacrylate (HEMA, 97%, MW = 130.14 g/mol), 4-cyano-4-(phenylcarbonothioylthio)pentanoic acid (CPA, ≥97%, MW = 279.38 g/mol), 4,4′-azobis(cyanovaleric acid) (ACVA, ≥98%, MW = 280.2 g/mol), Rhodamine B (≥95%, MW = 479.01 g/mol), Methacryloyl Chloride (MAC, ≥97%, MW = 104.53 g/mol), Phenol (MW = 94.11 g/mol), Potassium Tert Butoxide (≥98%, MW = 112.21 g/mol), 4 Chloro 3 Nitropyridine (90%, MW = 158.54 g/mol), Diethyl Ether (≥99.7%, MW = 74.12 g/mol), Sodium Sulphate (≥99.0%, MW = 142.04 g/mol), Hydrochloric acid (≥99.8%, MW = 36.46 g/mol), Stannous Chloride (98%, MW = 189.62 g/mol), Sodium hydroxide solution (50% in H_2_O, MW = 40.00 g/mol), Salicylaldehyde (≥98%, MW = 122.12 g/mol), Potassium carbonate (≥99%, MW = 138.21 g/mol), Propargyl bromide solution (80% in toluene, MW = 118.96 g/mol), Sodium borohydride (MW = 37.83 g/mol), Sodium bicarbonate (≥99.7%, MW = 84.01 g/mol), Hexane (95%, MW = 86.18 g/mol), Acetyl chloride (98%, MW = 78.50 g/mol), Triethylamine (TEA, ≥99.5%, MW = 101.19 g/mol), 4-(Dimethylamino)pyridine (DMAP, ≥99.9%, MW = 122.17 g/mol), 2-methacryloyloxyethylphosphorylcholine (MPC, 97%, MW = 295.27 g/mol), Ethanol (EtOH, ≥99.8% MW = 46.07 g/mol), Dimethyl Sulfoxide (DMSO, MW = 78.13 g/mol), Methanol (MeOH, MW = 32.04 g/mol), Acetonitrile (ACN, MW = 41.05), Dichloromethane (DCM, ≥99.9%, MW = 84.93 g/mol) and DCM anhydrous (dried over molecular sieves), Tetra Deuterated Methanol (MeOD-d4, ≥99.8%, MW = 36.07 g/mol), Deuterated Chloroform (CDCl_3_, 99.8%, MW = 120.38 g/mol), Sodium Sulphate (Na_2_SO_4_, ≥99.5%, MW = 82.03 g/mol), Ethyl Acetate (MW = 88.11 g/mol), Toluene (99.8% MW = 92.14 g/mol), Dimethylformamide (DMF, MW = 73,09 g/mol), Tris[(1-benzyl-1H-1,2,3-triazol-4-yl)methyl]amine (TBTA, MW = 530.63 g/mol) were purchased from Sigma Aldrich and used as received unless specifically noted.

All reagents and solvents were purchased from commercial sources and used without any further purification. The reactions were carried out under atmospheric air unless otherwise indicated, such as moisture-sensitive ones, for which a static nitrogen atmosphere was used. The reactions were monitored mostly by thin-layer chromatography (TLC), performed on Merck Kieselgel 60 F254 plates. Visualization was accomplished by UV irradiation at 254 nm and subsequently by treatment with the alkaline KMnO_4_ reactant or with a phosphomolybdic reagent. When necessary, crude compounds were purified through silica gel column chromatography (230–400 mesh). The NMR spectra were recorded on a Bruker 400 spectrometer (^1^H NMR, 400 MHz; ^13^C NMR, 100 MHz). The spectra were registered at room temperature in CDCl_3_ or MeOD-d4, unless otherwise indicated, with tetramethylsilane (TMS, δ = 0.0 ppm) used as the internal standard. The chemical shifts are reported as δ values in parts per million (ppm) in comparison to the internal standards; the coupling constants J are reported in Hz. The following abbreviations were used for spin multiplicity: s = singlet, d = doublet, t = triplet, dd = double doublet, m = multiplet, br s = broad singlet. Electrospray ionization mass spectrometry (ESI-MS) analyses were conducted on an Esquire 3000 Plus spectrometer, using methanol as a solvent. FT-IR spectrometers analysis were conducted on a Varian 640-IR FT-IR spectrometer.

### 2.2. Synthesis of 3-Nitro-4-phenoxypyridine (***1***)

**Scheme 1:**
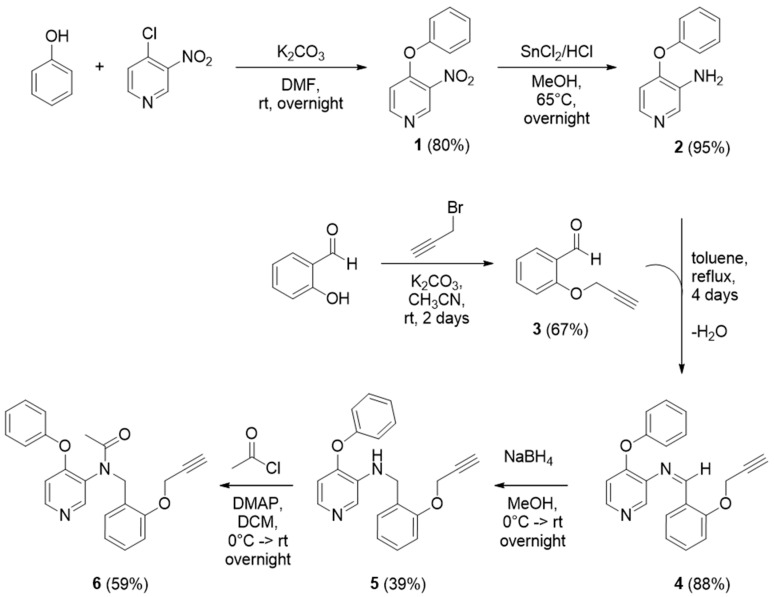
Synthesis of a modified PBR28 TSPO-ligand functionalized with a clickable alkyne group.

For the synthesis of nitro-4-phenoxypyridine, **1** in Scheme 1, 4.0 g of phenol (1.3 eq., 42.5 mmol) dissolved in 20 mL of DMF were added to a solution of 7.7 g (1.8 eq., 55.8 mmol) of potassium carbonate in 25 mL of DMF at room temperature. After 10 min, 5.0 g (1 eq., 31.5 mmol) of 4-chloro-3-nitropyridine in 25 mL of DMF were slowly added to the solution under magnetic stirring. The mixture was continuously stirred overnight at room temperature. The product was then extracted three times with diethyl ether (3 × 50 mL), washed with brine (3 × 50 mL), and dried over anhydrous sodium sulphate. After filtration, product **2** was recovered by evaporating the solvent at reduced pressure. The orange solid obtained (5.5 g, 80% yield) was analyzed through ^1^H-NMR (400 MHz, Chloroform-*d*) δ 9.11 (s, 1H), 8.53 (d, *J* = 5.9 Hz, 1H), 7.53–7.45 (m, 2H), 7.37–7.31 (m, 1H), 7.17–7.12 (m, 2H), 6.78 (d, *J* = 5.8 Hz, 1H).

### 2.3. Synthesis of 4-Phenoxy-3-pyridinamine (***2***)

For the synthesis of 4-phenoxy-3-pyridinamine **2**, 2.8 g of **1** (1 eq., 13 mmol) were dissolved in 20 mL of methanol and added to a solution of 15 g (6.8 eq., 79 mmol) of SnCl_2_ in 16 mL of 6 N HCl. The mixture was then refluxed overnight at 65 °C. After that, the product was concentrated at reduced pressure in order to remove the methanol. The pH of the residue was increased to 13 with 6 N NaOH and then the precipitated Sn(OH)_2_ was filtered off over celite, washing with ethyl acetate (100 mL). After reducing the solvent volume with a rotavapor, the product was extracted three times with ethyl acetate/NaHCO_3_ (aq) (3 × 50 mL). The organic layer was dried over anhydrous sodium sulphate, filtrated and evaporated at reduced pressure to give the resulting product **2** as a pure yellow solid (2.3 g, 95% yield), which was analyzed through ^1^H-NMR (400 MHz, Chloroform-*d*) δ 8.15 (s, 1H), 7.89 (d, *J* = 5.4 Hz, 1H), 7.45–7.36 (m, 2H), 7.25–7.19 (m, 1H), 7.11–7.05 (m, 2H), 6.58 (d, *J* = 5.4 Hz, 1H), 4.3 (bs, 2H).

### 2.4. Synthesis of 2-(Prop-2-yn-1-yloxy)benzaldehyde (***3***)

To a stirring solution of 3.0 mL of salicylaldehyde (1 eq., 30 mmol) in acetonitrile (80 mL), 10.37 g of potassium carbonate (2.5 eq., 75 mmol) were added. The mixture was stirred at room temperature and then 2.76 mL of propargyl bromide (1.1 eq., 31 mmol) were added, allowing it to react for 2 days. After that, the mixture was filtered, the liquid extracted three times with DCM/water (3 × 50 mL) and the organic residue was dried over anhydrous sodium sulfate. The solvent was removed under reduced pressure to obtain the brown pure solid **3** (3.21 g, 67% yield). The final product was analyzed through ^1^H-NMR (400 MHz, Chloroform-*d*) δ 10.47 (s, 1H), 7.85 (dd, *J* = 7.7, 1.8 Hz, 1H), 7.56 (ddd, *J* = 8.3, 7.3, 1.8 Hz, 1H), 7.11 (dd, *J* = 8.5, 0.9 Hz, 1H), 7.08 (tt, *J* = 7.5, 0.9 Hz, 1H), 4.82 (d, *J* = 2.4 Hz, 2H), 2.58 (t, *J* = 2.4 Hz, 1H).

### 2.5. Synthesis of 4-Phenoxy-N-(2-(prop-2-yn-1-yloxy)benzylidene)pyridin-3-amine (***4***)

To synthesize 4-phenoxy-*N*-(2-(prop-2-yn-1-yloxy)benzylidene)pyridin-3-amine **4**, a mixture of 2.8 g of **3** (1 eq., 17 mmol) and 3.17 g of **2** (1 eq., 17 mmol) in 80 mL of toluene was refluxed for 4 days; a Dean-Stark trap was used to remove the water formed during the reaction. After 4 days, the solution was dried under reduced pressure and the crude, brownish solid **4** (4.9 g, 88% yield) is used directly in the next reduction step.

### 2.6. Synthesis of 4-Phenoxy-N-(2-(prop-2-yn-1-yloxy)benzyl)pyridin-3-amine (***5***)

To synthesize 4-phenoxy-*N*-(2-(prop-2-yn-1-yloxy)benzyl)pyridin-3-amine **5**, 4.9 g of **4** (1 eq., 14.9 mmol) were dissolved in methanol (50 mL) and cooled to 0 °C in an ice bath. Then, 2.26 g of sodium borohydride (4 eq., 60 mmol) were slowly added to the solution, the ice bath was removed, and the mixture stirred overnight at room temperature. The methanol was removed under reduced pressure, then the crude was extracted three times with NaHCO_3_(aq) saturated solution/DCM (3 × 50 mL). The organic layers were combined and dried over anhydrous sodium sulfate, and then the product was recovered by removing the solvent with a rotavapor. The resulting crude (4.79 g) was a yellow oil, purified with a Flash Chromatography silica column, starting with 100% hexane, then increasing the polarity until 100% ethyl acetate and in the end switching to a mixture EA:MeOH (7:3). The fractions were collected and analyzed by TLC. The recovered pure product **5** (2.33 g, 39% yield) was a yellow oil. ^1^H-NMR (400 MHz, Chloroform-*d*) δ 8.10 (s, 1H), 7.86 (d, *J* = 5.3 Hz, 1H), 7.42–7.32 (m, 3H), 7.27 (ddd, *J* = 9.2, 6.7, 1.7 Hz, 1H), 7.23–7.16 (m, 1H), 7.10–7.03 (m, 2H), 7.04–6.94 (m, 2H), 6.55 (d, *J* = 5.3 Hz, 1H), 4.73 (d, *J* = 2.4 Hz, 3H), 4.49 (d, *J* = 5.8 Hz, 2H), 2.50 (t, *J* = 2.4 Hz, 1H).

### 2.7. N-(4-Phenoxypyridin-3-yl)-N-(2-(prop-2-yn-1-yloxy)benzyl)acetamide (***6***)

To a solution of pure **5** (2.33 g, 1 eq., 7.0 mmol) in DCM (50 mL), 2.6 g of DMAP (3 eq., 21.0 mmol) were added; this mixture was stirred for 5 min, then was cooled down to 0 °C in an ice bath. A solution of 1.5 mL of acetyl chloride (3 eq., 21.0 mmol) in DCM (5 mL) was added dropwise. The mixture was stirred under the ice for 90 min, then it was left stirring at room temperature overnight. The day after, the crude was extracted three times with NaHCO_3_(aq) saturated solution/DCM (3 × 50 mL). The organic layers were combined and dried over anhydrous sodium sulfate, then the product was recovered by removing the solvent with a rotavapor. The crude (2.1 g) was a brown oil, purified with a Flash Chromatography silica column, starting with hexane: ethyl acetate (7:3), then increasing the polarity until 100% ethyl acetate. The fractions were collected and analyzed by TLC. The recovered pure product **6** (1.54 g, 59% yield) was a yellow oil. ^1^H-NMR (400 MHz, Chloroform-*d*) δ 8.28 (d, *J* = 5.6 Hz, 1H), 8.19 (s, 1H), 7.41 (td, *J* = 7.2, 1.8 Hz, 3H), 7.30–7.18 (m, 3H), 6.95–6.89 (m, 3H), 6.87 (dd, *J* = 8.3, 1.0 Hz, 1H), 6.57 (d, *J* = 5.6 Hz, 1H), 5.20 (d, *J* = 14.2 Hz, 1H), 4.87 (d, *J* = 14.2 Hz, 1H), 4.46 (dd, *J* = 8.3, 2.4 Hz, 2H), 2.41 (t, *J* = 2.4 Hz, 1H), 1.98 (s, 3H).

### 2.8. Synthesis of the Methacrylamide-PEG-N_3_ Monomer

Amine-PEG-N_3_ (0.5 g, 0.775 mmol) and TEA (0.153 g, 1.51 mmol) were dissolved in 10 mL of anhydrous DCM. A solution containing 120 mg (1.14 mmol) of methacryloyl chloride in 0.5 mL DCM was added to the previous solution over 10 min under nitrogen atmosphere. The mixture was stirred at room temperature under a nitrogen atmosphere overnight. The solvent was then evaporated, the residue was dissolved in 50 mL of chloroform, washed with 50 mL of HCl 1 M, and then with 50 mL of brine. The organic layer was dried with anhydrous Na_2_SO_4_, filtered and evaporated to give the PEG-N_3_ monomer (MePEG-N_3_) as a transparent viscous liquid (0.47 g, yield 85%). The NMR spectrum of the molecule is reported in the Supporting Material (Figure S5).

^1^H-NMR (400 MHz, Chloroform-*d*) δ 6.5 (s, 1H), 5.88 (s, 1H), 5.28 (s, 1H), 3.38–3.65 (m, 44H), 1.96 (s, 3H).

### 2.9. Synthesis of the 25MPC-mMePEG-N_3_ Macromolecular Chain Transfer Agent

The RAFT copolymerization of MPC and MePEG-N_3_ was performed in a 40/60 *v/v* mixture of ethanol/acetic buffer with CPA as RAFT agent and ACVA as initiator. The MPC and ACVA to CPA mole ratios were set to 25 and 1/3, respectively. On the other hand, two MePEG-N_3_/CPA mole ratios, i.e., 4 and 6, were adopted to vary the number of azide groups. To synthesize the 25MPC-6MePEG-N_3_, where 25 and 6 represent the target degree of polymerization for the two repeating units MPC and MePEG-N_3_, respectively, 0.5 g of MPC, 0.322 g of MePEG-N_3_, 0.02 g of CPA and 0.0065 g of ACVA were dissolved in ethanol and acetic buffer and the solution was poured in a round bottom flask. The solution was purged by bubbling nitrogen for 20 min and then heated to 65 °C in a thermostatic oil bath for 24 h. Aliquots of the product were withdrawn before the purification and analyzed via ^1^H-NMR and aqueous gel permeation chromatography (A-GPC). Monomer conversion (*X_MPC_*) and the degree of polymerization (n) were evaluated via ^1^H-NMR spectroscopy dissolving 10 mg of the polymer in 0.6 mL of CD_3_OD-d4.

The number-average molecular weight (*Mn*) and dispersity (*Đ*) were evaluated via A-GPC with a Jasco 2000 chromatograph. A total of 10 mg of sample were dissolved in 2 mL of a 0.05 M Na_2_SO_4_/acetonitrile (80/20 *v*/*v*) mixture and filtered through a 0.45 μm pore-size nylon membrane. The separation was performed at a flow rate of 0.5 mL min^−1^ at 35 °C with three Suprema columns (particle size 10 μm and pore sizes of 100, 1000, and 3000 Å, Polymer Standards Service). All the values reported were determined from differential refractive index data and were relative to polyethylene glycol standards. The final product was precipitated several times in acetone and dried under air.

### 2.10. Synthesis of the Oligo(Caprolactone) Macromonomer

The oligo(caprolactone) macromonomer with 5 ε-caprolactone repeating units, hereinafter HEMA-CL_5_, was synthesized via ROP of CL in bulk with HEMA as chain initiator and Sn(Oct)_2_ as catalyst. The HEMA to Sn(Oct)_2_ molar ratio was set equal to 200 while the monomer to initiator molar ratio was set to 5. In particular, 13.15 g of CL and 13.2 mg of Na_2_SO_4_ were weighted in a round-bottom flask and heated to 125 °C in a constant temperature oil bath under stirring. A total of 3 g of HEMA and 56.7 mg of Sn(Oct)_2_ were stirred until homogenization and injected into the pre-heated round bottom flask. The reaction was allowed to proceed for 2.5 h. The final product was analyzed through ^1^H-NMR (CDCl_3_) and GPC (10 mg/mL in THF). Caprolactone conversion (*X_CL_*) and the average number of caprolactone units attached to HEMA (*q*) were evaluated via ^1^H-NMR while macromonomer number-averaged molecular weight (*Mn_GPC_*) and dispersity (*Đ_GPC_*) were evaluated via GPC. More in detail, the sample was dissolved in THF at 4 mg mL^−1^ and filtered through a PTFE 0.45 μm pore-size membrane before injection. The separation was performed at a flow rate of 0.5 mL min^−1^ at 35 °C and with three styrene/divinylbenzene (SDV) columns in series (Polymer Standard Service, Germany; pore size 10^3^, 10^5^, and 10^6^ Å; 300 mm length and 8 mm internal diameter) and a pre-column (50 mm length and 8 mm internal diameter). All the values reported were determined from differential refractive index data and were relative to poly(styrene) standards (from 580 to 3,250,000 g/mol, Polymer Laboratories).

### 2.11. Synthesis of the Amphiphilic and Biodegradable Block Copolymers

A set of amphiphilic block copolymers were obtained via RAFT polymerization of HEMA-CL_5_ in the presence of the zwitterionic-PEGylated macromolecular chain transfer agent synthesized previously and using ACVA as initiator. The ACVA to 25MPC-mMePEG-N_3_ molar ratio was set equal to 1/3, and the HEMA-CL_5_ to 25MPC-mMePEG-N_3_ molar ratio (*p*) was set to 30 or 60. As an example, for the 25MPC-6MePEGN_3_-30CL5, with 30 units of HEMA-CL_5_ added to the copolymer, 0.27 g of 25MPC-6MePEGN_3_, 0.52 g of HEMA-CL_5_ and 2.3 mg of ACVA were dissolved in 4 mL of methanol and poured in a 5 mL vial. The mixture was purged with nitrogen for 10 min and left to react at 65 °C under stirring for 24 h. The final block copolymer was precipitated in diethyl ether several times, dried under vacuum, and stored at −20 °C. An aliquot of the mixture was analyzed before and after the purification via ^1^H NMR (10 mg were dissolved in a mixture of 0.7 mL of MeOD). Macromonomer conversion (*X*) and the degree of polymerization (*pNMR*) were evaluated from the ^1^H NMR spectra.

### 2.12. Block Copolymer Functionalization with the TSPO-Ligand

The N-(4-phenoxypyridin-3-yl)-N-(2-(prop-2-yn-1-yloxy)benzyl)acetamide was conjugated to the block copolymer through click chemistry. Briefly, 0.05 g of 25MPC-6MePEGN_3_-30CL5 were added to 0.011 g of N-(4-phenoxypyridin-3-yl)-N-(2-(prop-2-yn-1- yloxy)benzyl)acetamide and 1.5 mg of TBTA. The mixture was solubilized in 0.3 mL of a solution of DMSO:MeOH (1/3 *v/v*). Finally, 0.5 mg of CuI were added to the flask and the final mixture was left to react at 37 °C for 3 days. The final product was analyzed through Fourier Transformed Infrared spectroscopy (FT-IR) to evaluate the intensity of the azide peak before and after the conjugation.

### 2.13. Docking Studies

The crystal structure of TSPO co-crystallized with PK11195 was retrieved from Protein Data Bank (PDB ID: 4RYI), prepared and used to dock ligands into the binding site. Both PBR28 and the derivative bearing the triazole ring formed after click chemistry (PBR28-triazole) were docked according to the following procedure. Ligand docking was carried out with Glide, a grid-based algorithm implemented in Maestro Schrödinger’s suite [18]. The grid was generated around the co-crystallized ligand with a radius of 15 Å. Default Van der Waals scaling was used, with a scaling factor of 1.0 and partial charge cut-off of 0.25. Partial charges were computed with the OPLS-2005 force field. Ligands were then docked into the receptor’s grid applying default scaling of Van Der Waals radii, flexible ligand sampling and standard or extra precision settings.

### 2.14. Nanoparticle Synthesis

The polymer NPs decorated with the developed TSPO-ligand were synthesized via self-assembly of the zwitterionic PCL-based block copolymer. Briefly, 10 mg of the PBR-functionalized polymer 25MPC-6MePEGPBR-30CL5 were solubilized in 50 μL of DMSO and 150 μL of MeOH and then added dropwise to 3 mL of distilled water under strong stirring. The NP dispersions were dialyzed in regenerated cellulose membranes (Spectra/Por, molecular weight cut-off = 3500 Da) against distilled water for 24 h to remove the organic solvent. The NP volume-average diameter (Dv) and polydispersity (PdI) were evaluated via dynamic light scattering (DLS) on a Zetasizer Nano ZS (Malvern, Instruments). Measurements were taken at a scattering angle of 173° and performed in triplicates on samples previously diluted to 0.1% *w*/*w* in distilled water.

### 2.15. Nanoparticle Staining

In order to visualize by fluorescent microscopy the internalization of NPs in cells, a fluorescent lipophilic dye was encapsulated. DiI (1,1′-Dioctadecyl-3,3,3′,3-tetramethylindocarbocyanine perchlorate, cat. # 468495, from Sigma-Aldrich, St. Louis, MO, USA) was used for this purpose. NPs (at 1 mg/mL in PBS) were incubated for 30 min at RT with DiI (0.2 mg/mL final concentration). Afterward, by taking advantage of the poorly water solubility of DiI and the high affinity of the NPs core for lipophilic compounds, the not encapsulated dye was removed from the solution by filtration through a 0.22 μm syringe methyl-cellulose filter.

### 2.16. In Vitro Tests

BV2 immortalized microglia cell line was used to assess the uptake of fluorescently labeled nanoparticles. BV2 cells were cultured at 37 °C, 5% CO_2_ with complete Dulbecco’s modified eagle’s medium (DMEM) supplemented with 10% heat-inactivated fetal bovine serum (FBS), 2 mM glutamine and 1% penicillin/streptomycin. To verify the endogenous expression and modulation of TSPO, BV2 cells were plated in 24-well tissue culture plates (15,000 cells/well). One day later, cells were treated for 24 h with LPS (from Sigma-Aldrich) at a final concentration of 0.05 or 1 μg/mL. Afterward, cells were detached with 0.05% Trypsin-EDTA, fixed with 4% phosphate-buffered paraformaldheyde (PFA) for 20 min and then stained with rabbit anti-TSPO (Abcam) antibody diluted 1:500 in BSA3% Triton 0.3% in PBS for 2 h at 4 °C. After one wash in PBS, cells were stained with anti-rabbit-Alexafluor-488 secondary antibody (Molecular Probes) at 1:1000 in BSA3% Triton 0.3% in PBS for 30 min at RT. TSPO signal was evaluated by flow cytometry with a BD Fortessa II cytometer. To visualize the mitochondria network, cells were plated on glass coverslips in 24 well plates and treated as described above. After the LPS treatment, cells were incubated for 15 min with Mitotracker-DR (Molecular Probes) at 150 nM at 37 °C in medium and then washed and fixed with 4% PFA. TSPO was stained following the same procedure applied for flow cytometry; nuclei were stained with DAPI 0.1 μg/mL in PBS for 10 min and then coverslips were mounted with Mowiol. The extent of co-localization between the mitochondria network and TSPO was analyzed at an SP5 laser scanning confocal microscope (Leica).

To highlight TSPO in live cells, lentiviral vectors expressing human TSPO (NM_000714) fused at the C-terminal with GFP reporter gene (from OriGene) were produced and titered as previously described [19]. These vectors were used to infect BV2 cells at MOI 5, to generate a cell line stably expressing TSPO-GFP fusion protein. Single-cell clones were generated by cell sorting and one clone expressing TSPO-GFP at high levels (hereafter called BV2-TSPO clone#4) was expanded and used for subsequent experiments.

For the assays, BV2-TSPO clone#4 cells were detached from the culture dishes, centrifuged at 150× *g* for 5 min and plated in 24-well tissue culture plates (15,000 cells/well) on glass coverslips. Twenty-four hours after plating, the medium was replaced with complete DMEM with different NP formulations (either not functionalized -6_30 NP- or modified by conjugation with PBR28-derivative -6PBR30 NP) at 5 μg/mL. At 4 h after NPs administration, the medium was removed and cells were washed with PBS and then fixed with 4% phosphate-buffered paraformaldheyde. Nuclei were stained with DAPI and then glass coverslips were mounted with Mowiol. High-resolution confocal microscope images were acquired at SP5 Leica confocal microscope equipped with UV-diode, Ne-He and Argon laser sources. No DiI signal was detected in cells incubated with the eluate obtained from the DiI diluted in PBS, confirming that no free DiI could be detected in the absence of nanoparticles upon filtration with 0.45 μm filter (data not shown).

Quantification of NPs internalization was performed by flow cytometry, by collecting the cells through trypsinization at 4 h after administration of NPs (at 1 μg/mL). The competition pharmacological assay consisted of co-incubation of NPs (either 6_30 or 6PBR30) tested at 1 μg/mL with 100 μM PBR28. Cells were analyzed by flow cytometry with a BD Facs Lyric cytometer.

## 3. Results

### 3.1. Docking Studies

**Figure 1:**
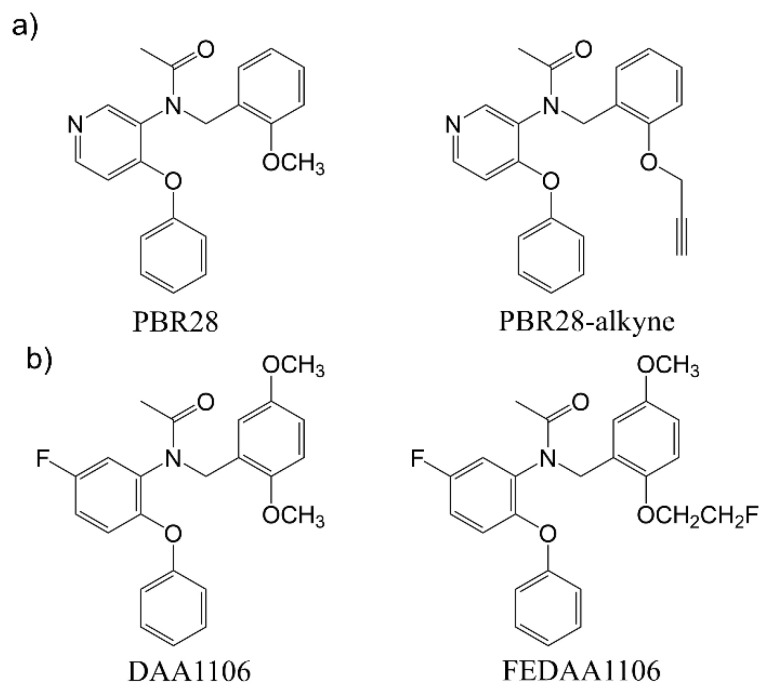
(**a**) Comparison of the molecular structure between PBR28 and the “clickable” PBR28-derivative synthesized in this work. (**b**) Molecular structures of two traditional TSPO ligands: DAA1106, FEDAA1106, PBR28 and the novel PBR28-alkyne.

The most critical hurdle hindering the suitability of PBR28 for conjugation with other molecules is the lack of easily exploitable reactive functional groups. We hypothesized that a PBR28-derivative, bearing a reactive alkyne functional group grafted on the methoxy group of the aromatic ring, could be a feasible strategy to allow the functionalization of PBR28 (PBR28-alkyne in Figure 1a). The alkyne group has the advantage of being reactive at mild conditions with an azide group forming 1,2,3-triazoles resistant to hydrolysis, thus preventing the risk of losing the targeting functionality upon exposure to biological fluids [13]. Moreover, the choice of the position of the alkyne group was based on the literature evidence suggesting that slight modifications of the O-substituent on the aromatic ring, such as in the case of DAA1106 and FEDAA1106 (Figure 1b), do not alter the selectivity for TSPO.

**Figure 2:**
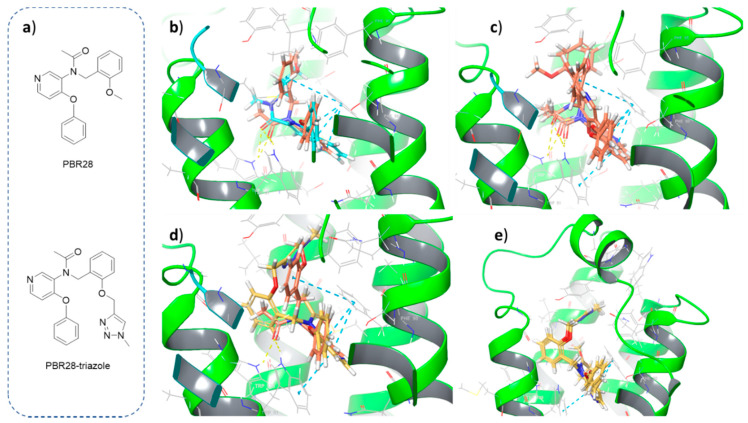
Chemical structures and docking poses of PBR28 and its derivative PBR28-triazole. Three-dimensional structures of ligands and key residues are shown as sticks. Hydrogen atoms are reported in white, oxygen in red and nitrogen in blue. H-bonds are depicted as yellow dotted lines, π–π stacking interactions are reported as blue dotted lines. (**a**) Two-dimensional structures of docked compounds, (**b**) superimposition of docked PBR28 (orange) with co-crystallized PK11195 (cyan), (**c**) superimposition of the best docking poses for PBR28, (**d**) superimposition of docked PBR28-triazole (yellow) with PBR28 (orange), (**e**) view of the best docking poses of PBR28-triazole.

To confirm that the triazole formed upon reaction of the alkyne moiety with the azide group does not interfere with binding with the receptor, we adopted an in silico approach using the TSPO crystal structure in complex with PK11195 (PDB ID: 4RYI) as a test platform to perform docking studies. First, PBR28 (Figure 2a, top) was docked into the binding site. The ligand occupies the same region as the co-crystallized compound and displays the same interactions with the key residues of the binding pocket, i.e., π–π stacking with Phe90 and H-bond with Trp51 and Trp138 (Figure 2b). Moreover, several similar poses were obtained for PBR28 (Figure 2c), all characterized by promising docking score values. These are obtained from the Glide scoring function, which describes the binding energy of the complex ligand-receptor [18]. In detail, the three best poses reported in Figure 2c displayed docking scores ranging from −9.154 to −9.432 (see Table 1). Taken together, these results indicate a good predictivity of the model. Hence, the obtained poses could serve as a template for evaluating the interactions of the new PBR28-derivative. Afterward, PBR28-triazole (Figure 2a, bottom) was docked into the TSPO binding site following the same procedure. This molecule was chosen as a model for the portion of the functionalized NP that should bind the target protein. In particular, it could give precious insights into the effects of the structural modification (i.e., the addition of a triazole ring) on the interactions with the binding site, indicating whether this is a viable approach or it could disrupt key interactions with the residues. As reported in Figure 2d,e, the new ligand occupies the binding site with a pose reasonably similar to the parent compound PBR28. The π–π stacking interactions with Phe90 are preserved, although minor rearrangement of the substituents is required to fit the additional triazole ring. Interestingly, different superimposable poses were obtained for PBR28-triazole, with the triazole moiety pointing toward the entrance of the binding pocket, in the direction of the solvent. Notably, the docking scores related to these poses range from −7.909 to −9.535 (see Table 1).

Overall, these results suggest the possibility of an optimal connection with the NP, without losing the most important interactions within the binding site.

### 3.2. Synthesis of Alkyne-Bearing TSPO-Ligand

The synthetic pathway of the modified ligand, PBR28-alkyne, is shown in Scheme 1. First, we synthesized the 3-nitro-4-phenoxypyridine (**1**) (structure and ^1^H-NMR spectrum in Figure S1) by addition of phenol to the commercially available 4-chloro-3-nitropyridine. The 4-phenoxy-3-pyridinamine (**2**) (structure and ^1^H-NMR spectrum in Figure S2) was obtained with high yields (i.e., 95%) via reduction of the NO_2_ group to an NH_2_ group, using HCl and Sn(Cl)_2_ as the catalyst. To provide the ligand with the desired alkyne group, the O-salicylaldehyde was functionalized with propargyl bromide to realize the 2-(prop-2-yn-1-yloxy)benzaldehyde (**3**) (structure and ^1^H-NMR spectrum in Figure S3). Combining compounds **2** and **3** we obtained the 4-phenoxy-*N*-(2-(prop-2-yn-1-yloxy)benzylidene)pyridin-3-amine (**4**) via formation of the Schiff’s base, promptly reduced with NaBH_4_ to give the 4-phenoxy-*N*-(2-(prop-2-yn-1-yloxy)benzyl)pyridin-3-amine (**5**) (structure and ^1^H-NMR spectrum in Figure S4). The further acetylation of **5** gave the final product *N*-(4-phenoxypyridin-3-yl)-*N*-(2-(prop-2-yn-1-yloxy)benzyl)acetamide (**6**) with an overall yield of 59%.

**Figure 3:**
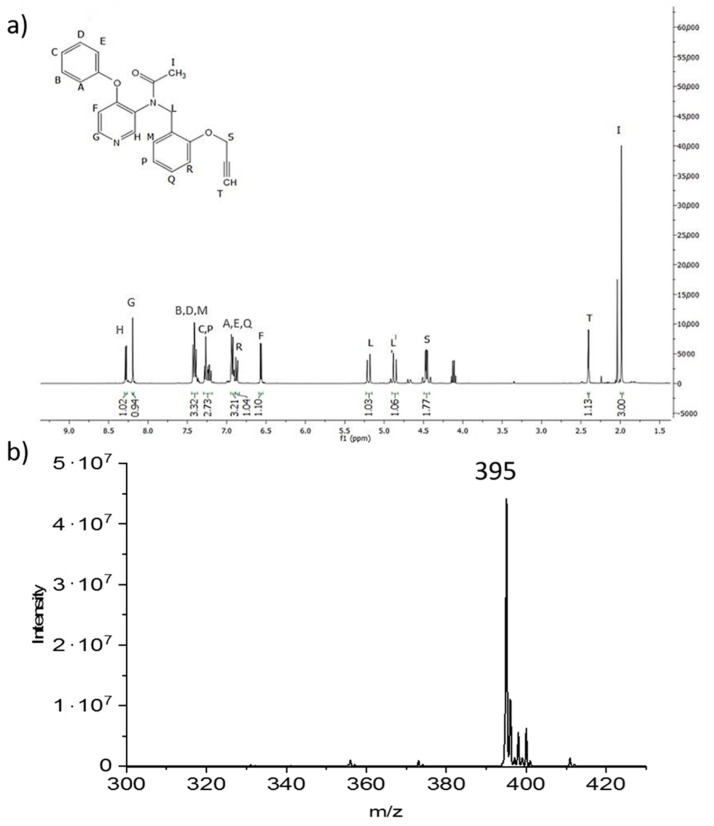
(**a**) ^1^H NMR of N-(4-phenoxypyridin-3-yl)-N-(2-(prop-2-yn-1-yloxy)benzyl)acetamide (PBR28-alkyne) (6) recorded in CDCl_3_ at 400 MHz. (**b**) Mass spectrum of the PBR28-alkyne confirming the correct synthesis of 6, with high purity.

The final product was analyzed via ^1^H NMR (Figure 3a) and ESI-MS (Figure 3b), both showing that the pure ligand with the desired alkyne functionalization was obtained. This opens the opportunity of using PBR28-alkyne to decorate via click chemistry polymer NPs exposing reactive azide groups on their surface.

### 3.3. NP Synthesis and Functionalization with the TSPO-Ligand

Recently, we reported the synthesis of zwitterionic biodegradable NPs whose properties can be readily modulated during the synthesis thus providing versatile platforms for the loading of poorly water-soluble drugs with minimal use of toxic organic solvents and providing enhanced stability to the formulation [17,20,21]. We showed that these NPs are able to be preferentially internalized in the microglia after administration into the spinal cord or in the cerebral lateral ventricles [20,22]. On the other hand, they were unable to differentiate between active and non-active microglia. The poor specificity did not allow us to track the course of neuroinflammation. Therefore, we now exploited the synthesized TSPO-ligand as a targeting agent for polymer NPs with the aim of improving their specific internalization in pro-inflammatory microglia.

In particular, the NPs are structurally composed of amphiphilic block copolymers. The hydrophilic portion of the block copolymers comprises zwitterionic units and is located on the NP surface, providing stability. Therefore, in order to maximize the exposure of the ligand to the TSPO, we decided to incorporate in this portion the azide functional groups necessary for the click reaction with the PBR28-alkyne. With this aim in mind, we synthesized polyethylenglycol-azide methacrylamide (MePEG-N_3_) via acylation of PEG-N_3_ with methacryloyl chloride (structure and ^1^H-NMR spectrum in Figure S5) and then we co-polymerized *m* units of this monomer with phosphoryl choline methacrylate via RAFT copolymerization to produce 25MPC-mMePEG-N_3_ macro RAFT agents. The use of RAFT polymerization, besides enabling to easily produce amphiphilic block copolymers, ensures low interchain composition drift and hence the proper incorporation of the azide functional monomer into each polymer chain [23]. We synthesized two macro RAFT agents, each comprising 25 units of MPC and *m* = 4 or 6 units of MePEG-N_3_, respectively, in order to test the influence of a different number of reactive azide groups and hence of TSPO-ligands on the final NPs (Table 2, structure and ^1^H-NMR spectrum in Figure S6).

On the other hand, the lipophilic portion of the block copolymer is synthesized from a biodegradable oligoester comprising 5 CL units and functionalized with a methacrylate group (HEMA-CL_5_, structure and ^1^H-NMR spectrum in Figure S7) and forms the NP core. Such oligoester macromonomer was produced through the ROP of CL using HEMA as initiator. In fact, in the ROP it is possible to choose a priori the number of CL units incorporated in the polymer chain by simply varying the stoichiometry between the monomer and the initiator [17]. The vinyl bond of HEMA gives us the possibility to further polymerize the macromonomer via RAFT polymerization. Therefore, we chain-extended the azide-containing macro RAFT agents with HEMA-CL_5_ targeting two degrees of polymerization (*p*), namely 30 and 60, in order to obtain two different amphiphilic block copolymers. These were analyzed via ^1^H-NMR and high monomer conversion and good control over *p* were confirmed (see Table 3 for properties and Figure S8 for the structure and ^1^H-NMR spectrum of one representative sample).

**Figure 4:**
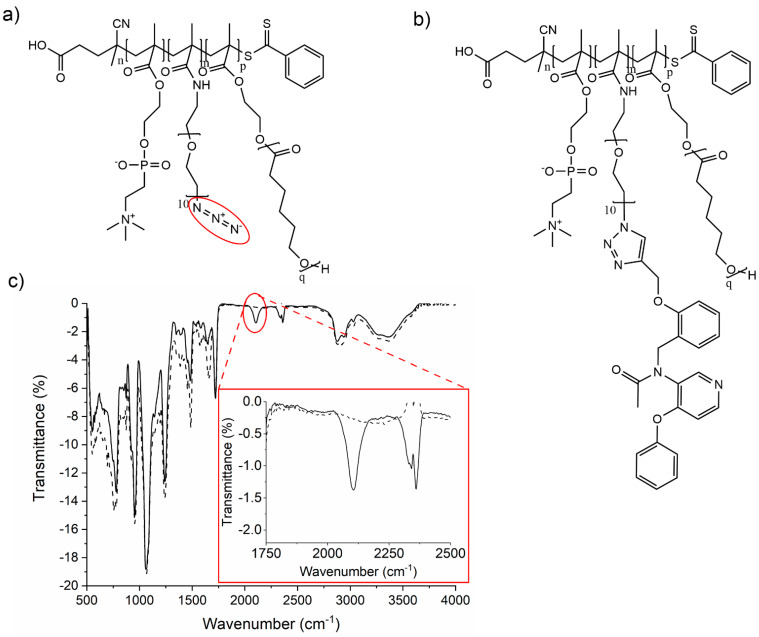
(**a**) Chemical structure of nMPC-mMePEGN_3_-pCLq, (**b**) Chemical structure of the functionalized polymer nMPC-mMePEGPBR-pCLq, (**c**) FT-IR spectra of the azide functionalized block copolymers 25MPC-6MePEGN_3_-30CL5 (solid line, entry 7 in Table 2) and of the product generated after the click reaction with PBR-alkyne, 25MPC-6MePEGPBR-30CL5 (dotted line, entry 3 in Table 2). The inset shows a magnification of the characteristic azide stretching band before (solid line) and after (dotted line) the conjugation with PBR-alkyne.

Finally, we functionalized the NPs with PBR28-alkyne via click chemistry. The final conjugation with the PBR28-alkyne was assessed by IR spectroscopy (Figure 4). The intensity of the characteristic vibration band of azide at 2100 cm^−1^ was found to be decreased and became almost invisible after clicking the TSPO-ligand, thus confirming that the reaction occurred properly.

The functionalized amphiphilic block copolymers and their non-functionalized counterparts used as control were finally nano precipitated in distilled water to form NPs (Table 3, entries 1–4 for the functionalized samples and entries 5–8 for the non-functionalized controls). As demonstrated in previous studies on similar materials, the adoption of oligoester-based repeating units to form the NP core ensures their degradation under physiological conditions and in turn the avoidance of polymer accumulation in the body [16,17,21]. At the same time, the average size of these colloids is in the range of 100–150 nm, confirming their suitability for systemic administration [15,24]. The functionalization with PBR28-alkyne was found to affect only slightly the NP size, which increased only by less than 10 nm going from the non-functionalized to the functionalized samples, while the morphology remained spherical, as judged from the transmission electron micrographs reported in Figure S9. On the other hand, the introduction of the PBR28 moiety had a significant impact on the particle size distribution, leading to its broadening, possibly due to an increased hindrance in the polymer chain diffusion and self-assembly. Among the produced samples, those carrying 6 units of MePEG-PBR (entries 3 and 4 in Table 3) are the ones with the narrowest distribution, which may be imputable to the steric stabilization introduced by the additional PEG chains. Therefore, we selected 6PBR30 NPs for the subsequent in vitro studies.

### 3.4. Internalization of PBR28-Functionalized NPs in BV2 Microglia-Like Cells

**Figure 5:**
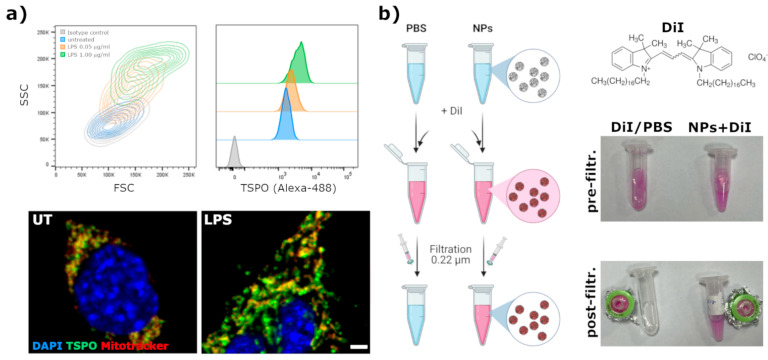
(**a**) Flow-cytometric and confocal fluorescence microscopy evaluation of TSPO expression and distribution in BV2 cells treated, or not, with LPS for 24 h. Scale bar = 2.5 μm; (**b**) Labeling procedure of NPs with the lipophilic dye DiI.

A BV2 murine microglia cell line was used for testing the uptake of TSPO ligand-conjugated NPs since the literature data reported the expression of TSPO in this cell line [25]. The experiments were carried out using the 6PBR30 batch (entry 3 in Table 3), as it is the one leading to the narrowest particle size distribution and suitable size for cell internalization studies. As shown in Figure 5a, we verified by flow cytometry the expression of TSPO in normal culture conditions and TSPO upregulation upon 24 h stimulation with LPS 1 μg/mL. Confocal microscopy highlighted a high co-localization between TSPO and mitochondria (evidenced by Mitotracker-DR); upon LPS stimulation, cell dimensions increase and the mitochondria network becomes more widely distributed in the cell cytoplasm, with a striking increase in TSPO signal density. To better visualize the pattern of internalization of NPs in the cells, we decided to generate a BV2 microglia cell line expressing a fluorescent TSPO. Briefly, we constructed a lentiviral vector plasmid carrying a ubiquitous promoter (hPGK) driving constitutive expression of human TSPO fused in frame, at the C-terminus, with GFP. This construct leads to the expression of a fluorescent human TSPO, allowing easy tracking of TSPO subcellular localization by fluorescent imaging microscopy. We used this cellular platform to monitor the internalization of PBR28-functionalized NPs and to study the extent of co-localization between these NPs and TSPO. The NPs were stained by encapsulating DiI. This is a highly lipophilic and poorly water-soluble fluorescent dye: hence, while it precipitates in PBS and can be easily removed by filtration, it could be efficiently encapsulated in the NPs (Figure 5b).

**Figure 6:**
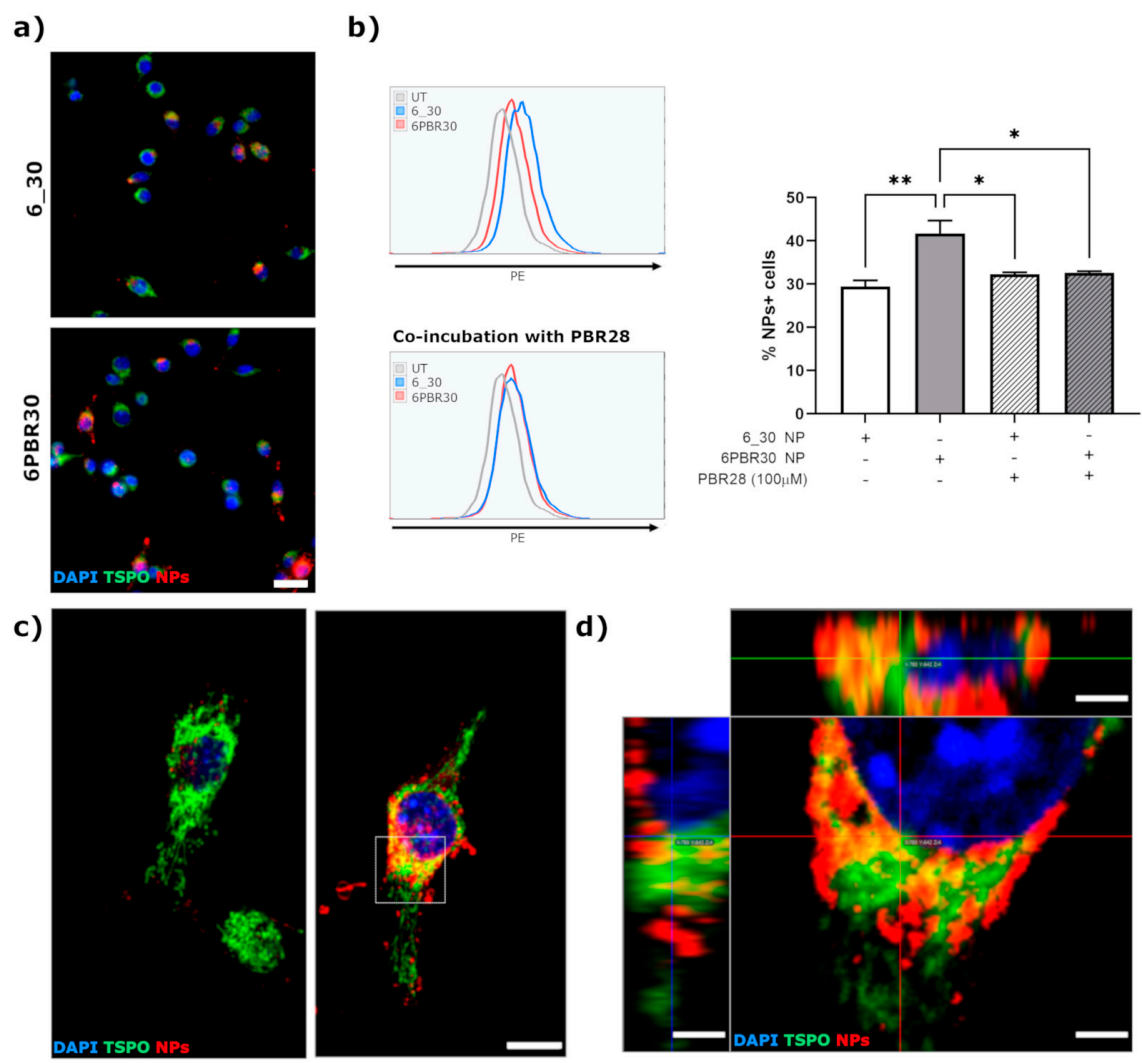
(**a**) Internalization of DiI-labeled 6_30 and 6PBR30 NPs in BV2 TSPO-GFP clone #4 cells, evaluated by epifluorescence microscopy at 4 h after NPs administration; scale bar = 25 μm; (**b**) Representative flow-cytometric plots and corresponding quantification of the percentage of DiI+ cells (measured in the phycoerythrin channel–PE) at 4 h after administration of DiI-labeled 6_30 or 6PBR30 NPs, in absence or presence of the free unconjugated PBR28 TSPO ligand. * = *p* < 0.05; ** = *p* < 0.01; ANOVA followed by Tukey’s post-hoc test; (**c**) representative laser scanning confocal microphotographs showing the extent of co-localization between NPs (red signal) and TSPO-GFP (green signal) at 4 h after NP administration, scale bar = 10 μm; (**d**) high magnification orthogonal projection of the region highlighted by the dashed-box in figure (**d**), scale bar = 2.5 μm.

DiI-labeled NPs were added to the cell cultures; then cells were washed in PBS and fixed with phosphate-buffered 4% PFA at 4h post-NP administration. As shown in Figure 6a, we could retrieve a more pronounced internalization of 6PBR30 in the cells compared to the non-functionalized 6_30 control (entry 7 in Table 3). This observation was confirmed through flow cytometry (as shown in Figure 6b) where we highlighted that 6_30 were internalized in about 30% of BV2 cells whereas with 6PBR30 this percentage significantly increased to 40%. To confirm the specificity of uptake for 6PBR30, we performed a competitive pharmacological assay by co-incubating the functionalized NPs with 100 μM of the free unconjugated TSPO ligand PBR28 for 4 h. As it can be observed from Figure 6b, while PBR28 did not affect the internalization of 6_30 in BV2 cells, the presence of the free unconjugated ligand significantly reduced the uptake of 6PBR30 suggesting that the internalization of these NPs in BV2 cells was at least in part mediated by the availability of free binding sites on the TSPO.

High-magnification pictures, obtained at the laser scanning confocal microscope, confirmed a partial co-localization of 6PBR30 with TSPO on the mitochondria network (Figure 6c,d). By comparison with the signal retrieved in BV2 cells exposed to unfiltered DiI (50 ng/mL) for 4 h, we confirmed that DiI remained actually encapsulated in the NPs after internalization in the cells; in fact, free DiI labels uniformly the cell membranes and organelles without showing the typical spot-like signal that we observed with DiI encapsulated in NPs (Figure S10).

## 4. Discussion and Conclusions

In this work, we successfully added a functional moiety to the high-affinity TSPO ligand PBR28 allowing for the generation of a PBR28-derivative suitable for conjugation with azide-functionalized polymer nanoparticles via click chemistry. First, we exploited in silico docking studies to exclude potential interferences with the binding of PBR28 to TSPO, caused by the triazole ring that is formed after the click reaction. Even if these analyses were not quantitative, we demonstrated that these in silico studies could be exploited as a guide for the rational design of small molecule putative TSPO-ligand candidates with optimal structural features. In fact, the good correlation that we observed between the poses of a reference TSPO-ligand, such as PK11195 (co-crystallized with the TSPO protein used for the docking studies) or PBR28 itself, and the PBR28-triazole derivative were successfully translated into experimental evidence of the suitability of the PBR28-alkyne, designed and synthesized in this study, for functionalizing the surface of polymeric NPs achieving efficient internalization in TSPO-expressing cells, such as BV2 microglia-like cell line.

Since its discovery in 1977 [26], radiolabeled ligands specific for TSPO were used in PET scans for many years to image the areas of enriched neuroinflammation in the diseased central nervous system of patients as well as rodent models of several neurodegenerative diseases, including Multiple Sclerosis, Alzheimer’s and Parkinson’s disease and ALS. A striking increase in TSPO expression was reported in several tumors, with a correlation with the level of malignancy, suggesting that TSPO ligands may be exploited also to identify cancerous cells and potentially target them with therapeutics. Accordingly, the development of bivalent compounds, derived from the conjugation of a TSPO ligand with a chemotherapic or a drug delivery system, is regarded as a promising strategy to achieve cell-selective targeting of therapeutics, testified by the increasing number of studies in the literature, reporting the exploitation of a diverse range of small molecule TSPO-ligands as a targeting moiety, ranging from TSPO-ligand chemotherapic (doxorubicin or cytarabine) [27,28] to TSPO-ligand dendrimers [9,29] or iron-oxide nanoparticle [7,30] conjugates. In line with these studies, in our work we demonstrated that the functionalization with a TSPO-targeted ligand enhances the internalization of polymer nanoparticles in TSPO-expressing cells; moreover, the internalized 6PBR30 NPs displayed a high degree of co-localization with the mitochondrial network. However, differently from other works where the mitochondria were highlighted, in our study we exploited a fluorescently tagged TSPO to obtain a more precise evaluation of the extent of co-localization between the PBR28-conjugated NPs and the target receptor. We observed that even if there are some regions in the cellular cytoplasm displaying good co-localization between the NP signal and TSPO, there are some NP signals that do not co-localize. This is not surprising and is in line with what has been reported by other authors (where only partial co-localization between TSPO-targeted NPs and mitochondria was reported [9,30]). In previous studies, we reported that our polymeric NPs can be internalized in microglia cells even without surface functionalization, through the interaction with scavenger receptors that are highly expressed by microglia/macrophages [22]. Based on the results of the confocal analyses performed with 6PBR30, and since TSPO is mainly localized on mitochondria, it can be hypothesized that the 6PBR30 NPs (as most likely also other TSPO-ligand functionalized NPs), are retained on the mitochondria network through the interaction of TSPO after cellular internalization through other mechanisms. This could represent an interesting mechanism by which TSPO-targeting could be exploited to enhance nanoparticle-mediated drug delivery to the mitochondrial compartment. Recent studies have highlighted increased pharmacological potency of apocynin (an inhibitor of NOX2, an enzyme involved in reactive oxygen species production) when targeted to mitochondria [31]. Interestingly, TSPO and the ER-resident NOX2 subunit gp91phox co-localize in primary microglia, pointing to a possible interaction between TSPO and NOX2 in these cells.

A polymorphism in the TSPO gene, evidenced in about 10% of the population, affects the binding of PBR28 [1]. Even if this could be envisaged as a possible limitation that could hinder the widespread applicability of the nanoparticle platform developed in our study, PBR28 is still widely used as a PET tracer (especially for tracking of neuroinflammation in neurodegenerative disorders) thanks to high affinity for the receptor and optimal biodistribution profile. So, exploiting PBR28-derivatives such as the one generated in this study, albeit only after the genetic screening of the patients to exclude poor binders, could represent an additional tool exploitable for the generation of cell- (including mitochondria-) selective targeted therapeutics.

## 5. Patents

R.A., M.S., U.C.P., D.M. and M.P. are authors on a patent related to the use of functionalized nanoparticles filed by Boston Children’s Hospital and Politecnico of Milano (WO 2019/191650 A1, filed on 29 March 2019).

## Figures and Tables

**Table 1:** Glide docking scores were obtained with Maestro for the top four poses of each ligand docked on TSPO.

Compound	Docking Score	Compound	Docking Score
PBR28	−9.432	PBR28-triazole	−9.535
	−9.240		−8.454
	−9.154		−8.286
	−8.934		−7.909

**Table 2:** Characteristics of the hydrophilic macro RAFT agents and of the macromonomer obtained via ROP. Mn_GPC_ = number-averaged molecular weight obtained from GPC, Ð_GPC_ = polymer dispersity, q_NMR_ = degree of polymerization of the hydrophobic macromonomer (i.e., average number of repeating CL units) derived from NMR, n_NMR_ = degree of polymerization of MPC obtained from NMR, m_NMR_ = degree of polymerization of MePEG-N_3_ obtained from NMR, χ = monomer conversion from NMR.

Sample	Mn_GPC_ [g/mol]	Ð_GPC_ [-]	q_NMR_ [-]	n_NMR_ [-]	m_NMR_ [-]	χ [%]
25MPC-4MePEG-N_3_	7206	1.18	-	23.7	3.8	97%
25MPC-6MePEG-N_3_	8140	1.23	-	23.7	5.4	98%
HEMA-CL_5_	754	1.29	5.4	-	-	98%

**Table 3:** Nanoparticle characterization in terms of degree of polymerization of the hydrophobic portion as determined q_NMR_ via ^1^H-NMR (p_NMR_), volume-average diameter (Dv) and polydispersity (PdI) as obtained through dynamic light scattering measurements.

Entry	Name	Block Copolymer	p_NMR_	Dv	PdI
			[-]	[nm]	[-]
1	4PBR30	25MPC-4MePEGPBR-30CL5	28	107	0.193
2	4PBR60	25MPC-4MePEGPBR-60CL5	54	128	0.255
3	6PBR30	25MPC-6MePEGPBR-30CL5	27	121	0.061
4	6PBR60	25MPC-6MePEGPBR-60CL5	51	139	0.130
5	4_30	25MPC-4MePEGN_3_-30CL5	28	104	0.038
6	4_60	25MPC-4MePEGN_3_-60CL5	54	110	0.114
7	6_30	25MPC-6MePEGN_3_-30CL5	27	118	0.047
8	6_60	25MPC-6MePEGN_3_-60CL5	51	101	0.114

## Data Availability

Data are fully available upon request.

## Supplementary Materials

The following are available online at https://www.mdpi.com/article/10.3390/nano11071693/s1, Electronic supplementary information are available at the publisher’s website and report the NMR spectra and characterization of all the intermediates of N-(4-phenoxypyridin-3-yl)-N-(2-(prop-2-yn-1-yloxy)benzyl)acetamide (PBR28-alkyne) synthesis, of the caprolactone-based macromonomer, PEGN_3_ methacrylamide, 25MPC and the block copolymer. Figure S1: ^1^H-NMR spectrum recorded in CDCl_3_ and at 400 MHz for 3-nitro-4-phenoxypyridine (1), Figure S2: ^1^H-NMR spectrum recorded in CDCl_3_ and at 400 MHz for 4-phenoxy-3-pyridinamine (2), Figure S3: ^1^H-NMR spectrum recorded in CDCl_3_ and at 400 MHz for 2-(prop-2-yn-1-yloxy)benzaldehyde (3), Figure S4: ^1^H-NMR spectrum recorded in CDCl_3_ at 400 MHz for 4-phenoxy-N-(2-(prop-2-yn-1-yloxy)benzyl)pyridin-3-amine (5), Figure S5: ^1^H-NMR spectrum recorded in CDCl_3_ and at 400 MHz for N-(2-(2-azidoethoxy)ethyl)methacrylamide, Figure S6: ^1^H-NMR spectrum recorded in CDCl_3_ and at 400 MHz for nMPC-m(MePEG-N_3_) with n = 25 and m = 6, Figure S7: Representative ^1^H-NMR spectrum of HEMA-CLq macromonomer with q = 5, Figure S8: ^1^H-NMR spectrum recorded in CDCl_3_ and at 400 MHz for nMPC-mMePEG-N_3_-pCL5 with n = 25, m = 6, and p = 30, Figure S9: TEM micrographs for: (a) 6_30 (entry 7 in Table 2) and (b) 6PBR30 (entry 4 in Table 2). Scale bar: 100 nm, Figure S10: Laser scanning confocal microphotographs showing the distribution of DiI signal in BV2 clone#4 TSPO cells exposed to unfiltered DiI 50 ng/mL for 4 h. Nuclei are stained with DAPI. Scale bar = 25 μm. The single channel showing DiI signal has been inverted to better highlight the distribution of the dye into the cell membranes.

## References

[1] Owen D.R., Yeo A.J., Gunn R.N., Song K., Wadsworth G., Lewis A., Rhodes C., Pulford D.J., Bennacef I., Parker C.A. (2012). An 18-kDa translocator protein (TSPO) polymorphism explains differences in binding affinity of the PET radioligand PBR28. J. Cereb. Blood Flow Metab..

[2] Alam M.M., Lee J., Lee S.Y. (2017). Recent Progress in the Development of TSPO PET Ligands for Neuroinflammation Imaging in Neurological Diseases. Nucl. Med. Mol. Imaging (2010).

[3] Kim S.W., Wiers C.E., Tyler R., Shokri-Kojori E., Jang Y.J., Zehra A., Freeman C., Ramirez V., Lindgren E., Miller G. (2018). Influence of alcoholism and cholesterol on TSPO binding in brain: PET [^11^C]PBR28 studies in humans and rodents. Neuropsychopharmacology.

[4] Casellas P., Galiegue S., Basile A.S. (2002). Peripheral benzodiazepine receptors and mitochondrial function. Neurochem. Int..

[5] Zarei S., Carr K., Reiley L., Diaz K., Guerra O., Altamirano P.F., Pagani W., Lodin D., Orozco G., Chinea A. (2015). A comprehensive review of amyotrophic lateral sclerosis. Surg. Neurol. Int..

[6] Lavisse S., Guillermier M., Hérard A.S., Petit F., Delahaye M., Van Camp N.V., Haim L.B., Lebon V., Remy P., Dollé F. (2012). Reactive astrocytes overexpress TSPO and are detected by TSPO positron emission tomography imaging. J. Neurosci..

[7] Iacobazzi R.M., Lopalco A., Cutrignelli A., Laquintana V., Lopedota A., Franco M., Denora N. (2017). Bridging Pharmaceutical Chemistry with Drug and Nanoparticle Targeting to Investigate the Role of the 18-kDa Translocator Protein TSPO. ChemMedChem.

[8] Bhoola N.H., Mbita Z., Hull R., Dlamini Z. (2018). Translocator Protein (TSPO) as a Potential Biomarker in Human Cancers. Int. J. Mol. Sci..

[9] Samuelson L.E., Anderson B.M., Bai M.F., Dukes M.J., Hunt C.R., Casey J.D., Han Z.Q., Papadopoulos V., Bornhop D.J. (2014). A self-internalizing mitochondrial TSPO targeting imaging probe for fluorescence, MRI and EM. RSC Adv..

[10] Briard E., Zoghbi S.S., Imaizumi M., Gourley J.P., Shetty H.U., Hong J., Cropley V., Fujita M., Innis R.B., Pike V.W. (2008). Synthesis and evaluation in monkey of two sensitive 11C-labeled aryloxyanilide ligands for imaging brain peripheral benzodiazepine receptors in vivo. J. Med. Chem..

[11] Kreisl W.C., Fujita M., Fujimura Y., Kimura N., Jenko K.J., Kannan P., Hong J., Morse C.L., Zoghbi S.S., Gladding R.L. (2010). Comparison of [11C]-(R)-PK 11195 and [^11^C]PBR28, two radioligands for translocator protein (18 kDa) in human and monkey: Implications for positron emission tomographic imaging of this inflammation biomarker. Neuroimage.

[12] Probst K.C., Izquierdo D., Bird J.L.E., Brichard L., Franck D., Davies J.R., Fryer T.D., Richards H.K., Clark J.C., Davenport A.P. (2007). Strategy for improved [^11^C]DAA1106 radiosynthesis and in vivo peripheral benzodiazepine receptor imaging using microPET, evaluation of [^11^C]DAA1106. Nucl. Med. Biol..

[13] Thirumurugan P., Matosiuk D., Jozwiak K. (2013). Click Chemistry for Drug Development and Diverse Chemical-Biology Applications. Chem. Rev..

[14] Hein J.E., Fokin V. (2010). V Copper-catalyzed azide-alkyne cycloaddition (CuAAC) and beyond: New reactivity of copper(I) acetylides. Chem. Soc. Rev..

[15] Ferrari R., Sponchioni M., Morbidelli M., Moscatelli D. (2018). Polymer nanoparticles for the intravenous delivery of anticancer drugs: The checkpoints on the road from the synthesis to clinical translation. Nanoscale.

[16] Sponchioni M., Bassam Rodrigues P., Moscatelli D., Arosio P., Capasso U. (2019). Biodegradable zwitterionic nanoparticles with tunable UCST-type phase separation under physiological conditions. Nanoscale.

[17] Capasso Palmiero U., Maraldi M., Manfredini N., Moscatelli D. (2018). Zwitterionic Polyester-Based Nanoparticles with Tunable Size, Polymer Molecular Weight, and Degradation Time. Biomacromolecules.

[18] Friesner R.A., Banks J.L., Murphy R.B., Halgren T.A., Klicic J.J., Mainz D.T., Repasky M.P., Knoll E.H., Shelley M., Perry J.K. (2004). Glide: A New Approach for Rapid, Accurate Docking and Scoring. 1. Method and Assessment of Docking Accuracy. J. Med. Chem..

[19] Capotondo A., Milazzo R., Garcia-Manteiga J.M.J.M., Cavalca E., Montepeloso A., Garrison B.S.B.S., Peviani M., Rossi D.J.D.J.D.J., Biffi A. (2017). Intracerebroventricular delivery of hematopoietic progenitors results in rapid and robust engraftment of microglia-like cells. Sci. Adv..

[20] Papa S., Rossi F., Ferrari R., Mariani A., De Paola M., Caron I., Fiordaliso F., Bisighini C., Sammali E., Colombo C. (2013). Selective Nanovector Mediated Treatment of Activated Proinflammatory Microglia/Macrophages in Spinal Cord Injury. ACS Nano.

[21] Auriemma R., Sponchioni M., Lotti S., Morosi L., Zucchetti M., Lupi M., Moscatelli D., Capasso Palmiero U. (2021). Preformed Biodegradable Zwitterionic Nanoparticles as Tunable Excipients for the Formulation of Therapeutics Directly at the Point of Care. Ind. Eng. Chem. Res..

[22] Peviani M., Capasso Palmiero U., Cecere F., Milazzo R., Moscatelli D., Biffi A. (2019). Biodegradable polymeric nanoparticles administered in the cerebrospinal fluid: Brain biodistribution, preferential internalization in microglia and implications for cell-selective drug release. Biomaterials.

[23] Sponchioni M., Capasso Palmiero U., Manfredini N., Moscatelli D. (2019). RAFT copolymerization of oppositely charged monomers and its use to tailor the composition of nonfouling polyampholytes with an UCST behaviour. React. Chem. Eng..

[24] Sponchioni M. (2020). Polymeric nanoparticles for controlled drug delivery. Nanomaterials for Theranostics and Tissue Engineering.

[25] Karlstetter M., Nothdurfter C., Aslanidis A., Moeller K., Horn F., Scholz R., Neumann H., Weber B.H.F., Rupprecht R., Langmann T. (2014). Translocator protein (18 kDa) (TSPO) is expressed in reactive retinal microglia and modulates microglial inflammation and phagocytosis. J. Neuroinflammation.

[26] Braestrup C., Alberchtsen R., Squires R.F. (1977). High densities of benzodiazepine receptors in human cortical areas. Nature.

[27] Jia J.B., Ling X., Xing M., Ludwig J.M., Bai M., Kim H.S. (2020). Novel TSPO-targeted doxorubicin prodrug for colorectal carcinoma cells. Anticancer Res..

[28] Denora N., Laquintana V., Trapani A., Lopedota A., Latrofa A., Gallo J.M., Trapani G. (2010). Translocator protein (TSPO) ligand-Ara-C (cytarabine) conjugates as a strategy to deliver antineoplastic drugs and to enhance drug clinical potential. Mol. Pharm..

[29] Sharma A., Liaw K., Sharma R., Thomas A.G., Slusher B.S., Kannan S., Kannan R.M. (2020). Targeting Mitochondria in Tumor-Associated Macrophages using a Dendrimer-Conjugated TSPO Ligand that Stimulates Antitumor Signaling in Glioblastoma. Biomacromolecules.

[30] Denora N., Lee C., Iacobazzi R.M., Choi J.Y., Song I.H., Yoo J.S., Piao Y., Lopalco A., Leonetti F., Lee B.C. (2019). TSPO-targeted NIR-fluorescent ultra-small iron oxide nanoparticles for glioblastoma imaging. Eur. J. Pharm. Sci..

[31] Dranka B.P., Gifford A.., McAllister D.., Zielonka J.J., Oseph J.., O’Hara C.L.., Stucky C.L.., Kanthasamy A.G.., Kalyanaraman B. (2014). A novel mitochondrially-targeted apocynin derivative prevents hyposmia and loss of motor function in the leucine-rich repeat kinase 2 (LRRK2(R1441G)) transgenic mouse model of Parkinson’s disease. Neurosci. Lett..

