# Supplementary material for: Synthesis and Characterization of a “Clickable” PBR28 TSPO-Selective Ligand Derivative Suitable for the Functionalization of Biodegradable Polymer Nanoparticles
Source: Nanomaterials (Basel). 2021 Jun 28;11(7):1693. doi: 10.3390/nano11071693 (PMC8308144; doi:10.3390/nano11071693)
Supplement: Supplementary file 1 [file nanomaterials-11-01693-s001.zip › nanomaterials-1197354-supplementary.pdf]

nanomaterials

Article

Synthesis and Characterization of a "Clickable" PBR28 TSPO-Selective Ligand Derivative Suitable for the Functionalization of Biodegradable Polymer Nanoparticles

Renato Auriemma [1,†], Mattia Sponchioni [1,*,†], Umberto Capasso Palmiero [2], Giacomo Rossino [3], Arianna Rossetti [1], Andrea Marsala [4], Simona Collina [3], Alessandro Sacchetti [1], Davide Moscatelli [1,†] and Marco Peviani [4,5,6,*,†]

[1] Department of Chemistry, Materials and Chemical Engineering "Giulio Natta", Politecnico di Milano, Via Mancinelli 7, 20131 Milano, Italy; renato.auriemma@polimi.it (R.A.); arianna.rossetti@polimi.it (A.R.); alessandro.sacchetti@polimi.it (A.S.); davide.moscatelli@polimi.it (D.M.)
[2] Department of Chemistry and Applied Biosciences, ETH Zürich, Vladimir-Prelog-Weg 1-5/10, 8093 Zürich, Switzerland; umberto.capasso@chem.ethz.ch
[3] Department of Drug Sciences, University of Pavia, Via Taramelli 12, 27100 Pavia, Italy; giacomo.rossino01@universitadipavia.it (G.R.); simona.collina@unipv.it (S.C.)
[4] Department of Biology and Biotechnology "L. Spallanzani", University of Pavia, Via Ferrata 9, 27100 Pavia, Italy; andrea.marsala01@universitadipavia.it (A.M.)
[5] Gene Therapy Program, Dana Farber/Boston Children's Cancer and Blood Disorders Center, 450 Brookline Ave., Boston, MA 02215, USA
[6] Harvard Medical School, Boston, MA 02115, USA
* Correspondence: mattia.sponchioni@polimi.it (M.S.); marco.peviani@unipv.it (M.P.)
† These authors contribute equally to this work.

Citation: Auriemma, R.; Sponchioni, M.; Capasso Palmiero, U.; Rossino, G.; Rossetti, A.; Marsala, A.; Collina, S.; Sacchetti, A.; Moscatelli, D.; Peviani, M. Synthesis and Characterization of a "Clickable" PBR28 TSPO-Selective Ligand Derivative Suitable for the Functionalization of Biodegradable Polymer Nanoparticles. *Nanomaterials* **2021**, *11*, x. https://doi.org/10.3390/xxxxx

Academic Editor(s): Pablo Botella; Christopher C. Landry; Suming Li

Received: 9 April 2021
Accepted: 22 June 2021
Published: date

Publisher's Note: MDPI stays neutral with regard to jurisdictional claims in published maps and institutional affiliations.

Copyright: © 2021 by the authors. Submitted for possible open access publication under the terms and conditions of the Creative Commons Attribution (CC BY) license (http://creativecommons.org/licenses/by/4.0/).

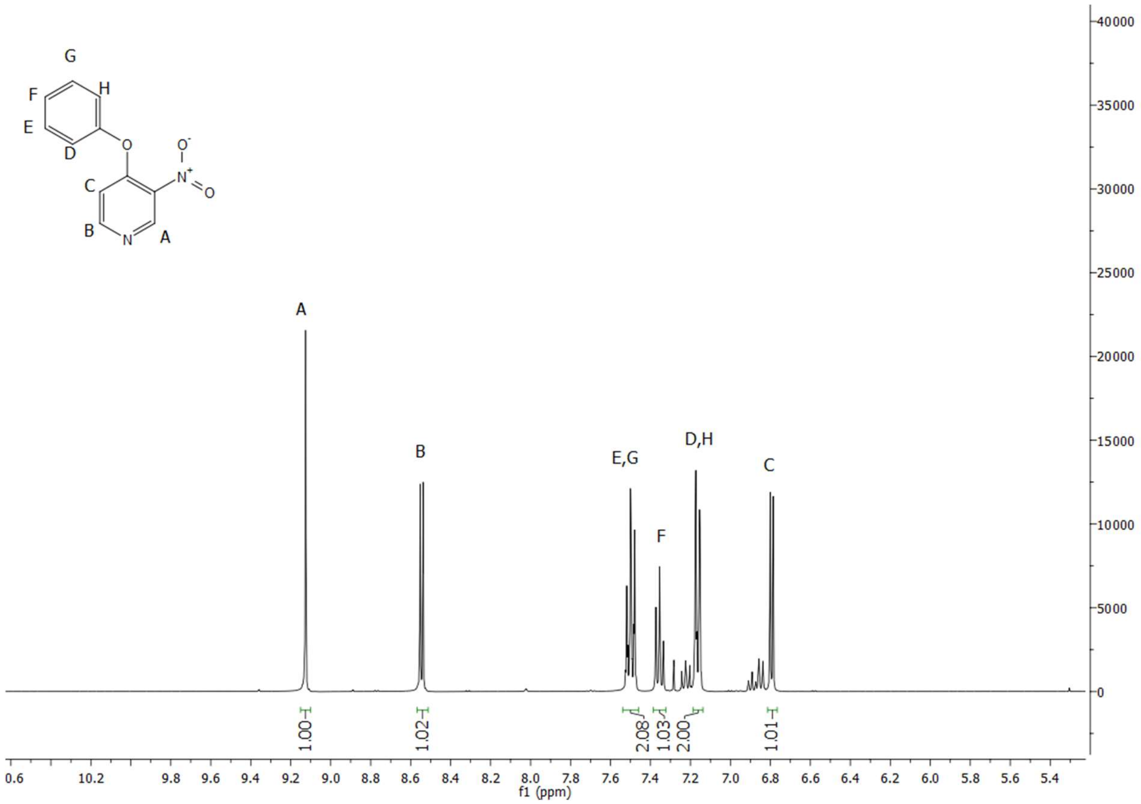

Figure S1. ^{1}H-NMR spectrum recorded in $CDCl_3$ and at 400 MHz for 3-nitro-4-phenoxypyridine (1).

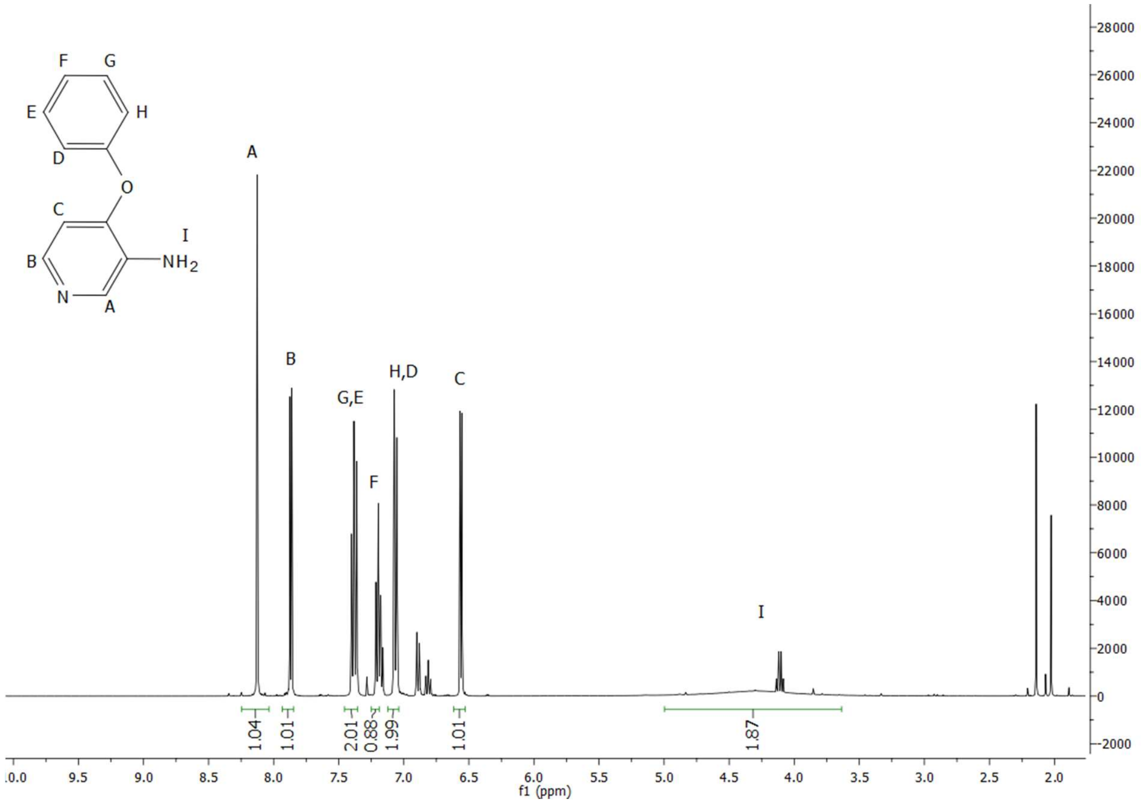

Figure S2. ^{1}H-NMR spectrum recorded in $CDCl_3$ and at 400 MHz for 4-phenoxy-3-pyridinamine (2).

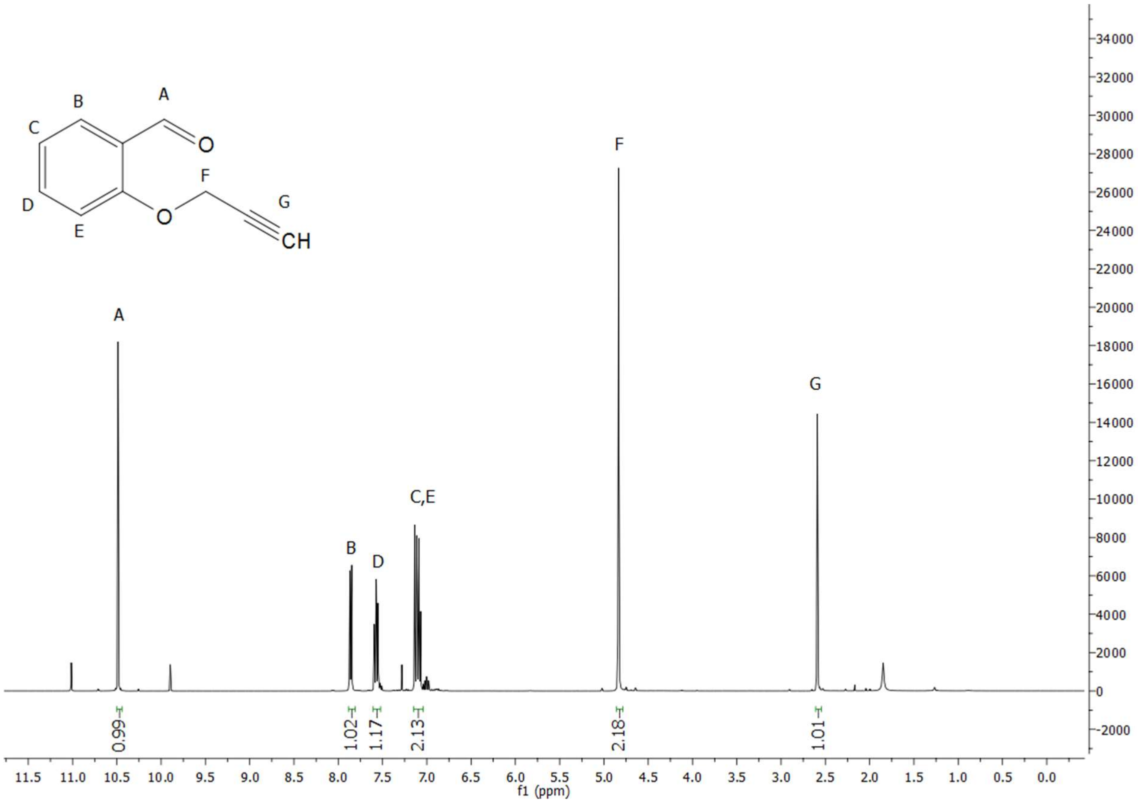

Figure S3. ^{1}H-NMR spectrum recorded in $CDCl_3$ and at 400 MHz for 2-(prop-2-yn-1-yloxy)benzaldehyde (3).

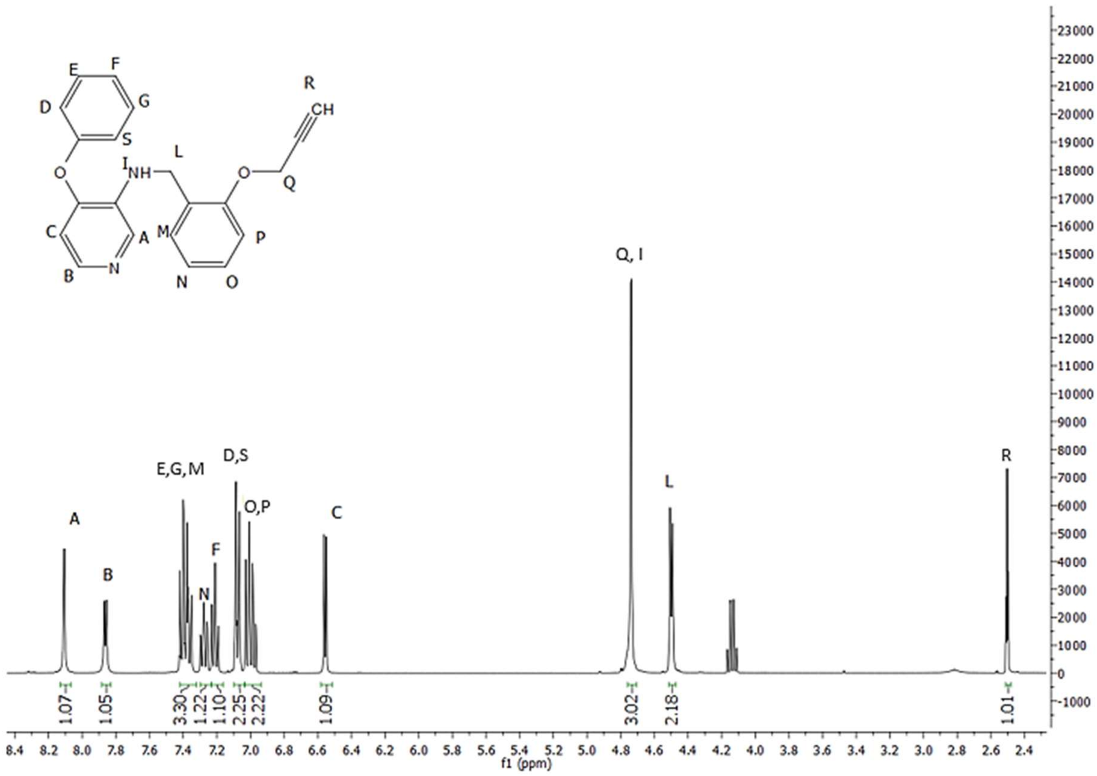

Figure S4. ^{1}H-NMR spectrum recorded in $CDCl_3$ at 400 MHz for 4-phenoxy-N-(2-(prop-2-yn-1-yloxy)benzyl)pyridin-3-amine (5).

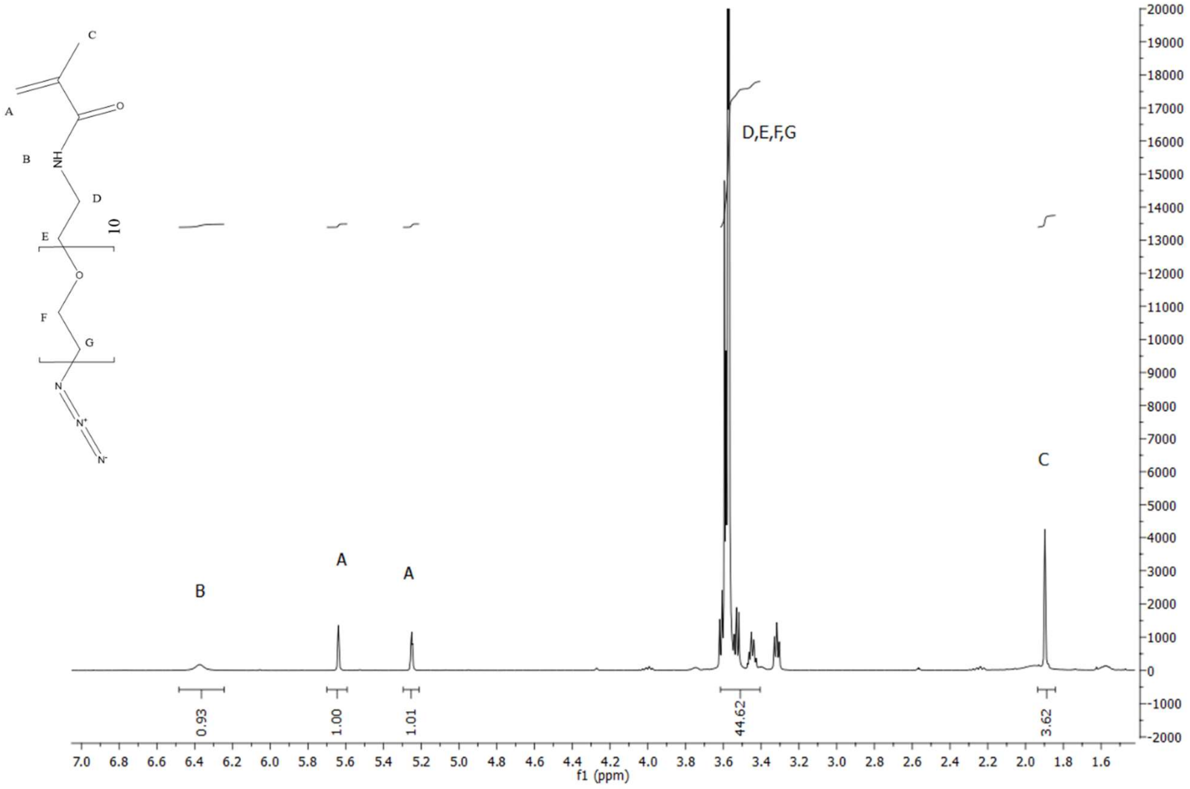

Figure S5. ^{1}H-NMR spectrum recorded in $CDCl_3$ and at 400 MHz for N-(2-(2-azidoethoxy)ethyl)methacrylamide.

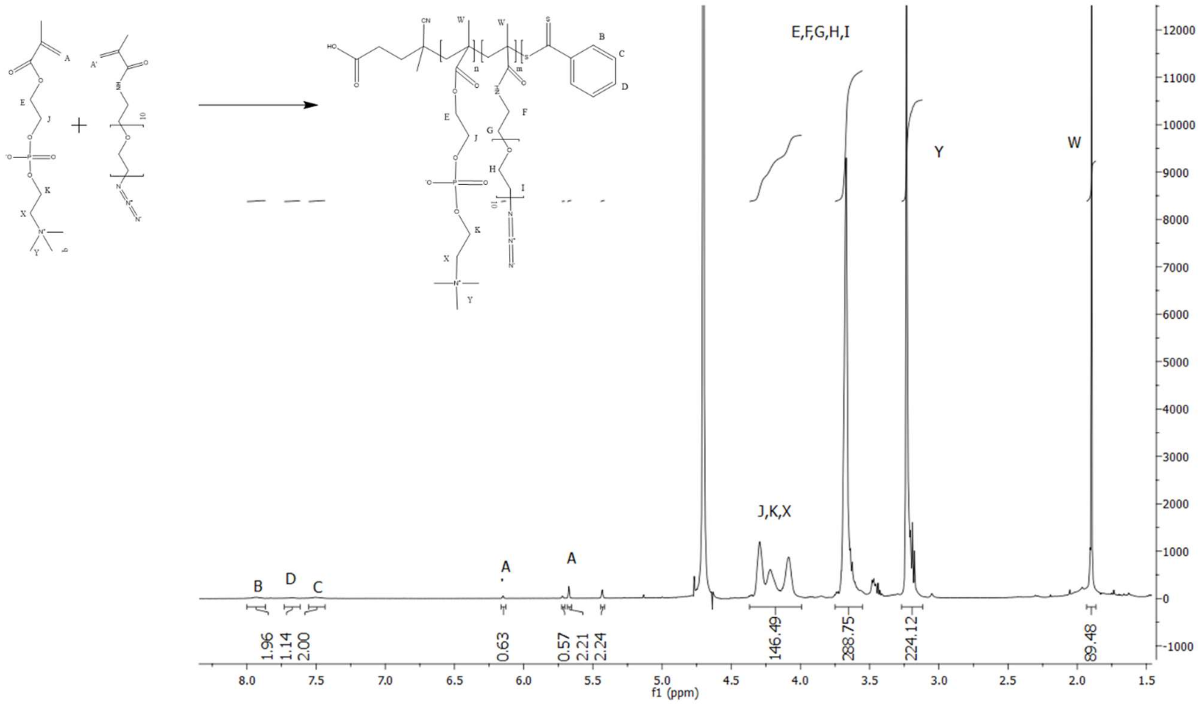

Figure S6. ^{1}H-NMR spectrum recorded in $CDCl_3$ and at 400 MHz for nMPC-m(MePEG-N_3) with n = 25 and m = 6.

The DP (n) of MPC and (m) of MePEG-N_3 in the macro RAFT agents nMPC-mMePEG-N_3 can be calculated as described in equations S1–S2:

$$n = \frac{[J,K,X]}{3 \times C} \times \chi_{MPC} \quad \text{(S1)}$$

$$m = \frac{[E,F,G,H,I] - 2 \times n}{22 \times C} \times \chi_{PEGN3} \quad \text{(S2)}$$

The peak C that belongs to CPA, was chosen as reference and its area was set equal to 2. To compute the DP, peaks J, K, X were employed as they belong to the 6 H of both the monomeric and polymerized MPC.

The conversion of MPC and MePEG-N_3 (χ_{MPC} and χ_{PEGN3}, respectively), were instead calculated according to equations S3–S4:

$$X_{MPC} = 1 - \frac{2 \times A}{J,X,K} \quad \text{(S3)}$$

$$X_{PEGN3} = 1 - \frac{2 \times A'}{[E,F,G,H,I] - 2 \times n} \quad \text{(S4)}$$

To calculate the monomer conversion, for the MPC the peak of one of the hydrogen atoms of the double bond (*A*), only present on the unreacted monomer, was compared to the peaks *J*,*X*,*K* corresponding to two hydrogen atoms each, referred to the converted polymer and unreacted monomer. For the MePEG-N_3 at the peak of [*E*,*F*,*G*,*H*,*I*] was subtracted the value of the peak of MPC corresponding to 2 hydrogens per unit of MPC and evaluated with the peak *A'*.

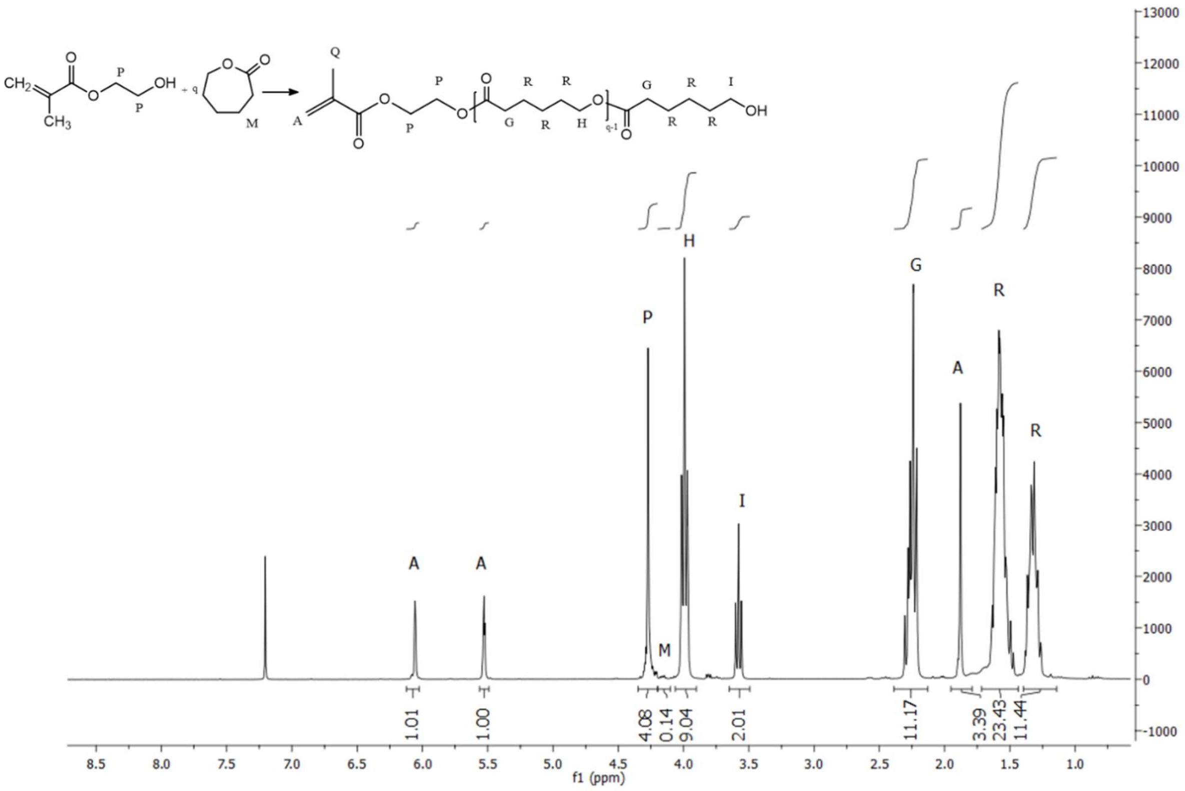

Figure S7. Representative ^{1}H-NMR spectrum of HEMA-CLq macromonomer with q = 5.

The DP (*q*) of HEMA-CLq is calculated as described in equation S5:

$$q = \frac{2(H + I)}{P} \quad \text{(S5)}$$

The peaks *I* and *H*, represent respectively the two hydrogen atoms adjacent to the chain-end hydroxyl group and to the ester group, while P refers to the 4 protons of the HEMA used as initiator.

The conversion X_{CL}, was calculated according to equation S6:

$$X_{CL} = \frac{H + I}{H + I + M} \quad \text{(S6)}$$

The peak M of the two hydrogens adjacent to the ester group of the monomeric CL was compared to the peaks H and I, of the two hydrogen atoms adjacent to the chain-end hydroxyl group and to the ester group of the polymerized CL, respectively.

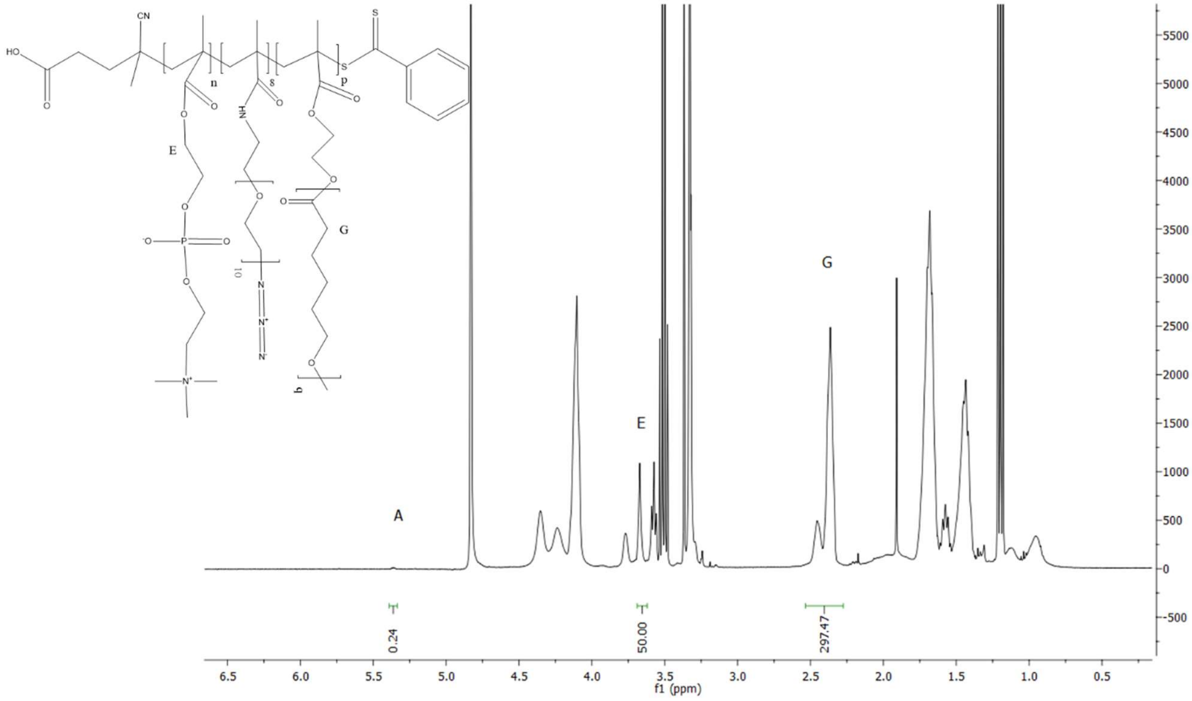

Figure S8. ^{1}H-NMR spectrum recorded in $CDCl_3$ and at 400 MHz for nMPC-mMePEG-N_3-pCL5 with n = 25, m = 6, and p = 30.

The peak E corresponding to the two hydrogen atoms present only in MPC was chosen as a reference and set equal to 50 (2 hydrogen atoms of the methylene group multiplied by 25 units of MPC).

The DP of HEMA-CL5 (p) can be calculated as described in equation S7:

$$p = \frac{n \times G}{5 \times E} \tag{S7}$$

The peak G refers to the two hydrogen atoms present in both reacted and unreacted macromonomer units, while 5 is the number of repeating caprolactone units in the macromonomer.

The macromonomer conversion $\chi_{HEMA\text{-}CL5}$ is determined according to equation S8:

$$\chi_{HEMA-CL5} = 1 - \frac{2 \times 5 \times A}{G} \tag{8}$$

The peak A, corresponding to one of the two hydrogen atoms of the lipophilic monomer double bond and the peak G were weighted on their number of hydrogens (2 H per 5 repeating units), and the final product was subtracted from 1.

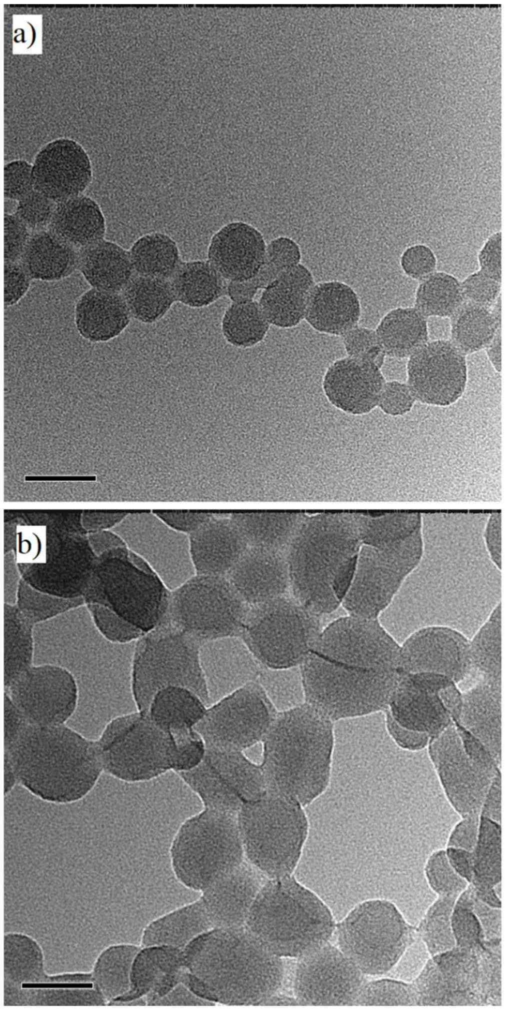

Figure S9. TEM micrographs for: (**a**) 6_30 (entry 7 in Table 2) and (**b**) 6PBR30 (entry 4 in Table 2). Scale bar: 100 nm.

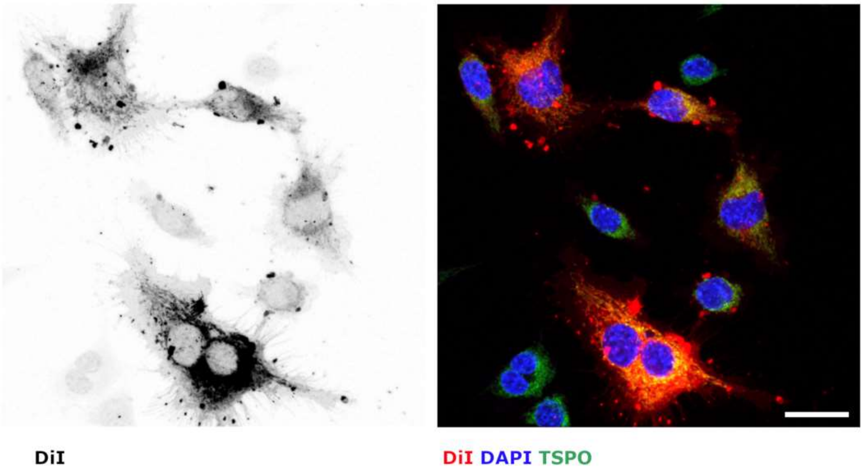

Figure S10. Laser scanning confocal microphotographs showing the distribution of DiI signal in BV2 clone#4 TSPO cells exposed to unfiltered DiI 50 ng/ml for 4h. Nuclei are stained with DAPI. Scale bar = 25 μm. The single channel showing DiI signal has been inverted to better highlight the distribution of the dye into the cell membranes.
